# COPII cage assembly factor Sec13 integrates information flow regulating endomembrane function in response to human variation

**DOI:** 10.1038/s41598-024-60687-2

**Published:** 2024-05-03

**Authors:** Frédéric Anglès, Vijay Gupta, Chao Wang, William E. Balch

**Affiliations:** Department of Molecular Medicine, The Scripps Research Institute, 10550 North Torrey Pines Rd, La Jolla, CA 92037 USA

**Keywords:** Biochemistry, Cell biology, Computational biology and bioinformatics, Genetics, Molecular biology, Physiology, Systems biology

## Abstract

How information flow is coordinated for managing transit of 1/3 of the genome through endomembrane pathways by the coat complex II (COPII) system in response to human variation remains an enigma. By examining the interactome of the COPII cage-assembly component Sec13, we show that it is simultaneously associated with multiple protein complexes that facilitate different features of a continuous program of chromatin organization, transcription, translation, trafficking, and degradation steps that are differentially sensitive to Sec13 levels. For the trafficking step, and unlike other COPII components, reduction of Sec13 expression decreased the ubiquitination and degradation of wild-type (WT) and F508del variant cargo protein cystic fibrosis transmembrane conductance regulator (CFTR) leading to a striking increase in fold stability suggesting that the events differentiating export from degradation are critically dependent on COPII cage assembly at the ER Golgi intermediate compartment (ERGIC) associated recycling and degradation step linked to COPI exchange. Given Sec13’s multiple roles in protein complex assemblies that change in response to its expression, we suggest that Sec13 serves as an unanticipated master regulator coordinating information flow from the genome to the proteome to facilitate spatial covariant features initiating and maintaining design and function of membrane architecture in response to human variation.

## Introduction

An understanding of the spatial covariant properties of information flow linking genotype to phenotype through transcription, translation, degradation, and endomembrane trafficking pathways^1–7^ is required for addressing the critical features regulating the genome to proteome transformation driving cell development, differentiation, and its response to aging and environmental stress. As part of this information flow system, an understanding of the spatial and temporal design of the coat complex II (COPII) system that distinguishes amongst > 8000 cargo destined for export from the endoplasmic reticulum (ER) and its response to human variation impacting protein folding in health and disease remains largely an enigma^8–15^. We have demonstrated that these events are part of an encompassing endomembrane ‘quality system (QS)’ designed to manage sequence-to-function-to-structure relationships dictated by the protein fold that can be quantitatively interrogated by a Gaussian process (GP) based spatial covariance (SCV) machine learning framework^1–7^. SCV reveals the intrinsic design features of the protein fold on a residue-by-residue basis at atomic resolution for the entire polypeptide sequence. SCV relationships provide detailed insight into the diverse roles of individual residues in the cell in the context of the spatial–temporal dynamics dictating endomembrane flow and function in health and disease^1–5,7^.

Although considerable progress has been made in unraveling the molecular basis of the basic pathways directing cargo flow through exocytic and endocytic compartments, how genome sequence information is transformed into endomembrane biology will require a deep understanding of the individual roles of trafficking machinery components. The first step in the exocytic pathway involves the assembly of COPII^10,16–32^. In vitro and in vivo studies have now shown that Sar1, Sec23, Sec24, Sec13 and Sec31 are cytosolic proteins that are required for COPII coat formation^8–10,25,27,32–37^. Together, they direct nascent cargo in the ER to all downstream exocytic, endocytic and ER-associated autophagic pathways^21,31,38–40^. Coat recruitment is initiated through the activation of Sar1 by the guanine nucleotide exchange factor (GEF) Sec12. The membrane recruitment and activation of Sar1 is followed by the binding of the cytosolic heterodimeric Sec23/24 complex. The Sec23 subunit is a Sar1-specific GTPase-activating protein (GAP) that has a catalytic role in GTP hydrolysis. By contrast, Sec24 serves as a multi-functional cargo-adaptor subunit that makes cargo choices for ER export. In general, the Sec23/24 complex functions as an kinetic adaptor platform for the selection of cargo for export that is sensitive to, for example, both di-acidic exit^41^ and basic retention codes^42^ as well higher order protein–protein interactions^10,18,19,22,28,43–47^. Subsequently, the Sec 13/31 heterotetramer complex is recruited to the nascent cargo-containing coat complex assembly^8,9,33,37,48–52^. Sec13/31 is currently thought of as a cage assembly factor to recruit cargo laden Sec23/24 complexes to form coated vesicles that bud from the ER for transit to the Golgi^8,9,33,37,53–56^. More recently, they are thought to contribute to the formation of collars at ER export sites (ERES) that are proposed to spatially regulate ‘cargo flow’ into tubules for direct trafficking to downstream ER Golgi intermediate compartments (ERGIC), Golgi and/or lysosomal compartments^25^.

While COPII dependent anterograde cargo export from the ERGIC provides a mechanism for export, it also initiates the recruitment of COPI coats where COPI directs retrograde recycling of misfolded cargo^51,57^, ER cargo receptors and ER-resident proteins such as the folding chaperone Hsp70 paralog BiP, by exchange of COPII for COPI^51,57^. COPI mediated recycling can lead to cargo degradation by the ERAD machinery^13,20,25,44,58–62^, an important step in the overall QS maintaining normal function of a cell^1–7^. Curiously, Sec13, a WD-repeat β-propeller family member, is also involved in nuclear pore complex (NPC) assembly directing flow of mRNA from the nucleus to the cytoplasm^63,64^, the GATOR2 complex^65–67^ that serves as a nutrient sensor through mTOR in the lysosomal pathway, as well as interacting with other WD-repeat containing proteins that regulate, for example, neuronal function leading to severe peripheral neuropathies when mutated^47^. The full-extent of Sec13 interactions in the cell remains unknown- nor is the coordination of Sec13 function between these different systems directing the composition and dynamics of endomembrane structures appreciated. These results raise the possibility that Sec13 may have a more fundamental role in controlling global information flow in the cell from the genome in response to diverse SCV relationships^1–7^.

As a model cargo protein engaged by COPII^3,4,7–9,33,34,41,50,51,68–70^, the cystic fibrosis (CF) transmembrane conductance regulator (CFTR) is a multi-membrane–spanning polypeptide belonging to the ATP binding cassette (ABC) transporter family^71,72^. It is composed of five functional domains: two nucleotide binding domains (NBD1 and NBD2), two trans-membrane domains (TMD1 and TMD2) and one regulatory domain^73–77^. The CFTR molecule is largely a cytosolic oriented protein with the regulatory ATP binding domains NBD1 and NBD2 required for export from the ER^4,78–80^.The biogenesis of CFTR requires trafficking from the ER, the first step in the exocytic pathway, through the Golgi to its final destination at the apical cell surface of epithelial cells in multiple tissues. CFTR functions as a cAMP-sensitive chloride channel at the apical plasma membrane (PM) of polarized epithelial cells^76–78^. It is charged with maintaining ion balance and hydration in, among others, sweat, intestinal, pancreatic, and pulmonary tissues^81^. Over 2000 variants contribute to human disease with the primary variant affecting 70–80% of the population having a deletion of residue Phe508 (F508del) leading to instability in the ER, degradation and loss of function at the cell surface^3,4,7,34,68,74,75,77,82–90^. The loss of a functional CFTR channel disrupts ion homeostasis, resulting in increased mucus viscosity in the airway of the lung and ductal systems of the pancreas and liver, and hydration of the intestinal tract. The increased mucus viscosity causes increased risk for inflammation and infection by *Pseudomonas aeruginosa* and other bacterial pathogens in the lung^81^. We have applied GP based SCV machine learning^1–7^ to achieve a deep understanding of the QS that dictates management of CFTR variation across the entire CF population to begin to understand as a collective the basis for precision management of disease in the individual^3,4,7^.

To address the global role of the COPII cage assembly component Sec13 in information flow through the endomembrane system via cargo selection into the COPII coat, we generated a Sec13 interactome using human bronchial epithelial (HBE) cells expressing wild-type CFTR, identifying 226 and 174 proteins significantly increased or decreased upon siRNA mediated Sec13 silencing, respectively. These changes potentially impact nucleopore complex (NPC) assembly, trafficking, the autophagy-lysosomal function through GATOR2 complex regulating mTORC1^65–67^, along with a number of unanticipated protein complexes that contribute to functionality of endomembrane systems in each cell type. We now show that siRNA Sec13 (siSec13) silencing dramatically (~ ten-fold) improves the stability WT CFTR for trafficking from the ER, leading to a decrease of both WT and F508del CFTR degradation. This striking change in stability was not observed in response to the silencing of other COPII components including Sar1a, Sec31, Sec23 and 24 isoforms. In conjunction with its additional roles in chromatin organization, transcription, translation, and autophagy, we now suggest that Sec13 based complex assembly serves as an unanticipated master regulator impacting information flow through endomembrane compartments^3,4^. By providing a pivotal ‘set-point’^7^ for diversion of traffic at the ERES-ERGIC interface through COPII dependent COPI exchange^9,34,49–51,57,70,91^, a step dictated by SCV relationships revealed by human variation^1–7^, Sec13 could have a central role in balancing global endomembrane function impacting health, disease and aging in response to the environment and natural selection.

## Results

### Sec13 interactome profile in human bronchial epithelial cells

**Figure 1:**
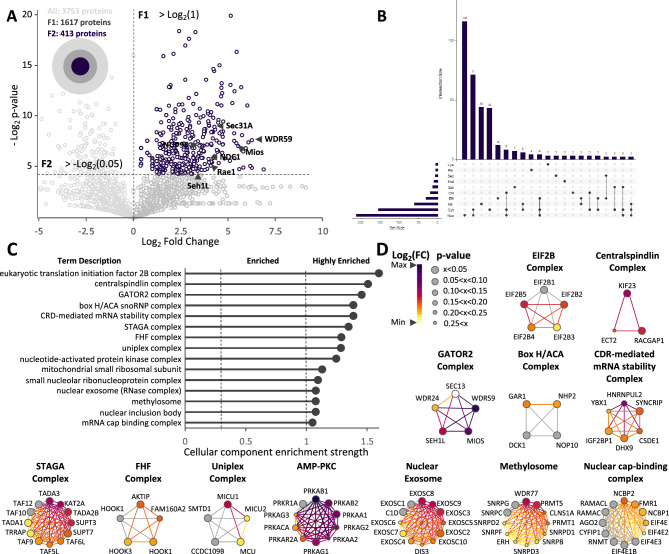
Sec13 core interactome. (**A**) Volcano plot of 3753 quantified proteins from HBE cells recovered by Sec13 IP compared to control IP displaying the relationship between statistical significance and fold-change of each protein. The fold-change is plotted on the x axis, and the − log_2_ p-value is plotted on the y axis. The blue circles represent the 413 proteins that had statistically significant differential interactions, log_2_ fold-change > 0 (p-value < 0.05). (**B**) UpSet plots showing the intersections of protein sets interacting with Sec13 present in the nucleus (Nuc), cytoplasm (Cyt), mitochondria (Mit), endoplasmic reticulum (ER), cell membrane (CM), Golgi apparatus (Gol), endosome (End), secreted (Sec), peroxisome (Per) and lysosome (Lys). The connected circles indicate which locations are included in the intersection. The horizontal bar chart displays the total number of proteins significantly interacting with Sec13 in a specific location. The vertical bar chart displays the number of proteins significantly interacting with Sec13 at that intersection. (**C**) The 413 proteins composing the Sec13 core interactome were mapped to the STRING database for cellular component analysis. 15 independent cellular components highly enriched (STRING enrichment strength > 1) were identified. (**D**) Networks of highly enriched complexes in the Sec13 interactome displaying the fold-change (yellow to purple) and the p-value (big to small circle) of the Sec13 interactome data set for each of the individual known components of these complexes. Gray circles represent complex subunits that were not identified in the Sec13 interactome.

To improve our understanding of COPII coat complex function in cargo traffic from the ER^3,4^, we first applied mass spectrometry (MS) to determine the Sec13 interactome given the central role of the Sec13/31 cage assembly complex in coordinating the assembly of Sec23/24 coat components that select newly synthesized cargo for maturation through exocytic pathway for function in downstream endomembrane compartments and at the cell surface^9,32,54,56,92^. This is the key step in the biology of the cell that controls information flow from the genome for nearly 1/3 of the proteome. For this purpose, we applied co-purifying protein identification technology (CoPIT), an immunoprecipitation (IP)-based proteomic-profiling approach for capturing protein–protein interactions coupled with multidimensional chromatography and MS^87^. Using a wild-type (WT) human bronchial (lung) epithelial (HBE) 41o- (HBE41o-) cell line harboring the COPII dependent cargo protein CFTR^3,4,7,34,68,87^, we recovered proteins mapping to 413 genes classified as high-confidence interactors (Fig. 1A, Supplementary Table S1) given the strong correlation observed among three biological replicates (Supplementary Fig. S1, Pearson’s r = 0.82–0.93). These 413 proteins form the core of the Sec13 interactome and include both direct and indirect interactions amenable to CoPIT recovery using a Sec13 specific antibody. An additional 1219 interactors with an increased fold-change over background, but with lower confidence scores, were further assembled into an extended interactome (Supplementary Table S2). All known interactors of Sec13 such as Sec31A of the COPII complex^29,33,37,52,93^, Nup98 and other components of the nuclear pore complex (NPC)^94–98^, as well as Mios, WDR24, WDR59 and Seh1L of the GATOR2 complex^65–67^, were identified, validating the CoPIT approach for discovery of additional Sec13 interactors (Fig. 1A).

In addition to the known interactions, we identified multiple interactors distributed along the exocytic and endocytic pathways (Fig. 1B, Supplementary Fig. S1). Interestingly, only Sec31A was found in the Sec13 interactome- not other components of the COPII coat complex, including any of the cargo selection Sec23/24 isoforms. These results highlight the expected strong interaction between Sec13 and Sec31A, but also emphasize the weak and likely transient interactions between the first layer of the COPII coat complex containing Sec23/24 cargo complexes and the second layer cage assembly Sec13/Sec31A complex leading to cargo collection and export from the ER, a state that is believe to be rapidly disassembled, coupled to COPI exchange^51,57^ to allow continued flow of cargo for recycling or to downstream endomembrane compartments^8,9,37,99^.

WD40 repeat domains, such as found in Sec13 are largely recognized to coordinate the assembly of multi-protein complexes^100–102^. To understand this role, we calculated the cellular enrichment of the Sec13 interactome components through STRING analysis tool^103^ (Fig. 1C, Supplementary Fig. S2). Here, we observed that all highly enriched components are multi-protein complexes (Fig. 1C, D) such as its known interactions facilitating NPC assembly^94–97^ to direct flow of mRNA from the nucleus to the cytoplasm^63,64^, and the GATOR2 complex^65–67^ that serves as a nutrient sensor in the mTORC1 lysosomal/autophagy pathway. In addition, we now show that it also includes the eukaryotic translation initiation factor 2B (EIf2B) complex^104^, the chromatin acetylating STAGA complex^105–107^ and the ubiquitous AMP-activated protein kinase complex (PKC), among many others (Fig. 1C, D; Supplementary Fig. S2). These results highlight a more general role of the steady state pool of Sec13 in managing multi-protein complex assemblies involved in the continuum of processes stemming from chromatin organization through transcription in the nucleus as well as co-translational insertion into the ER to generate a functional protein fold that can interact with trafficking components to initiate and maintain the composition and flow of secretory and membrane-bound cargo through the endomembrane system to the cell surface.

### Proteomic profiling of human bronchial epithelial cells treated with siSec13

**Figure 2:**
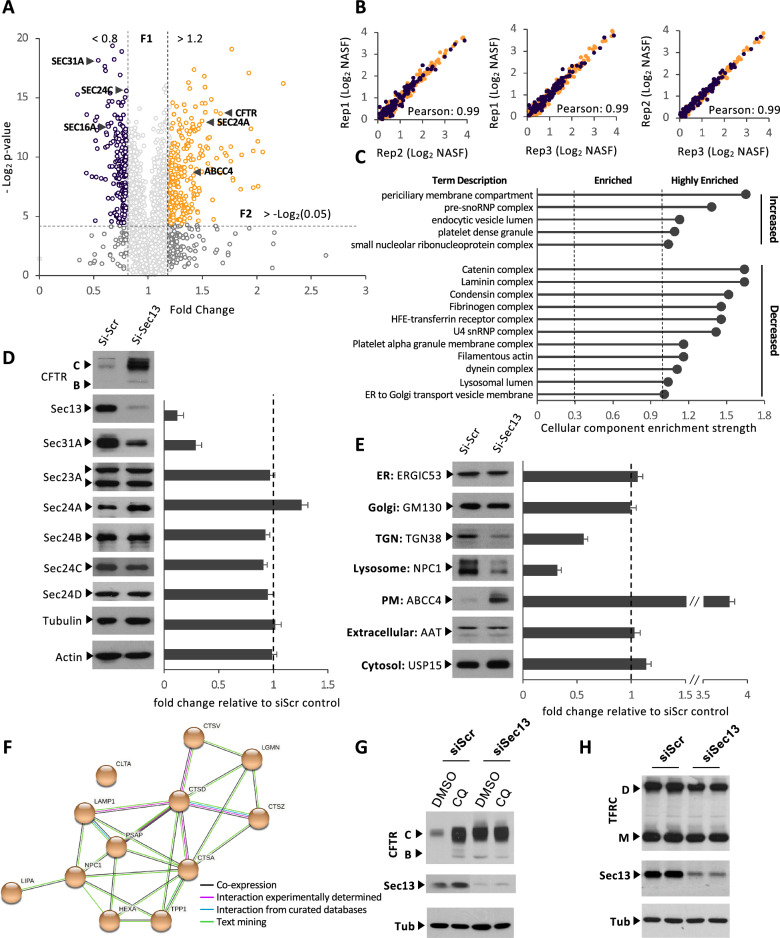
Proteome profiling following siRNA-mediated Sec13 silencing. (**A**) Volcano plot of 3603 quantified proteins from HBE41o- cells recovered following siRNA-mediated Sec13 silencing compared to siScr control displaying the relationship between statistical significance and fold-change of each protein. The fold-change is plotted on the x axis, and the − log_2_ p value is plotted on the y axis. The purple and orange circles represent the 184 and 241 proteins significantly decreased and increased upon Sec13 silencing, respectively (FC < 0.8 or FC > 1.2 with p-value < 0.05). (**B**) Scatterplot showing correlation of the NASF-scores among three replicates of quantified proteins recovered from HBE41o- cells following siRNA-mediated Sec13 silencing. (**C**) The 241 and 184 proteins significantly increased and decreased upon Sec13 silencing, respectively, were mapped to the STRING database for cellular component analysis. 5 and 11 cellular components are highly enriched among the proteins significantly increased and decreased upon Sec13 silencing, respectively. (**D**) Immunoblots (left panel) and quantifications of protein level (right panel) of individual COPII components following siSec13 treatment. Data are presented in right panel as fold-change relative to siScr control transfection (mean ± SEM, n ≥ 3). (**E**) Immunoblots (left panel) and quantifications of protein level (right panel) localized in different compartments of the cell following siSec13 treatment. Data are presented in right panel as fold-change relative to siScr control transfection (mean ± SEM, n ≥ 3). (**F**) Protein network of all lysosomal proteins that significantly decreased upon Sec13 silencing. (**G**) CFTR immunoblot of HBE41o- cells treated with DMSO or chloroquine following siRNA transfection with siSec13 or siScr. (**H**) Transferrin-receptor (TFRC) immunoblot of HBE41o- cells after siSec13 or siScr transfection. See Fig. S8 for full non-cropped blots used to generate figure.

To understand in more detail the role of Sec13 in the cell, we investigated the relative expression level of proteins in HBE41o- cells expressing WT CFTR that were treated with either siSec13 or a control scrambled siRNA (siScr) using a TMT-based isobaric labeling method followed by data acquisition using MudPIT^108^. siSec13 gives a nearly complete reduction of Sec13, although residual levels Sec13 may insure cell survival given that it is an essential protein^32,114,115^. We identified a total of 3603 distinct proteins, with 226 and 174 proteins significantly increased and decreased upon Sec13 silencing, respectively (Fig. 2A, Supplementary Table S3) with a high correlation between the different biological replicates (Fig. 2B, Pearson’s r = 0.99). These results suggest that loss of Sec13 can simultaneously either negatively or positively influence the stability of multiple pathways. Among the more stabilized pool, CFTR is the 19^th^ most increased protein upon Sec13 silencing (Fig. 2A, Supplementary Fig. S3, Supplementary Table S3) (see below).

We used the levels of differentially expressed proteins to calculate enrichment upon siSec13 treatment through the STRING analysis tool^103^ (Fig. 2A, C; Supplementary Fig. S3). We observed a significant increase in a large number of proteins in these cellular complexes, including components affecting ER to Golgi transport (Fig. 2A, Supplementary Fig. S3) and proteins localized to the lumen of the lysosome reflecting the role of Sec13 cage assembly in ER-associated autophagy-lysosome pathways (Fig. 2C)^21,31,38,39,47,66,109–113^. These results suggest a role for Sec13 in more globally managing protein fold stability (Fig. 1). To further refine the analysis, we compared the overlap between Sec13 interactors (Fig. 1A) to the changes in the global HBE41o- proteome following Sec13 silencing (Fig. 2; Supplementary Fig. S4, fold-change range < 0.8 or > 1.2), a cut-off to focus on more abundant interactors. This comparison revealed a common subset of Sec13-protein interactions affected by siSec13 silencing (Supplementary Fig. S4). As expected, we observed a prominent decrease in the Sec31A component involved in COPII cage assembly that requires Sec13 for stability^2,131–133^. We also observed changes in a number of Sec13 interactors found in the nucleus, ER, cytoplasm and in cytoskeletal organization. The combined results support a potential role for Sec13 in the stabilization and coordination of activities central to managing endomembrane function and structure.

### Effect of siSec13 on COPII mediated trafficking

**Figure 3:**
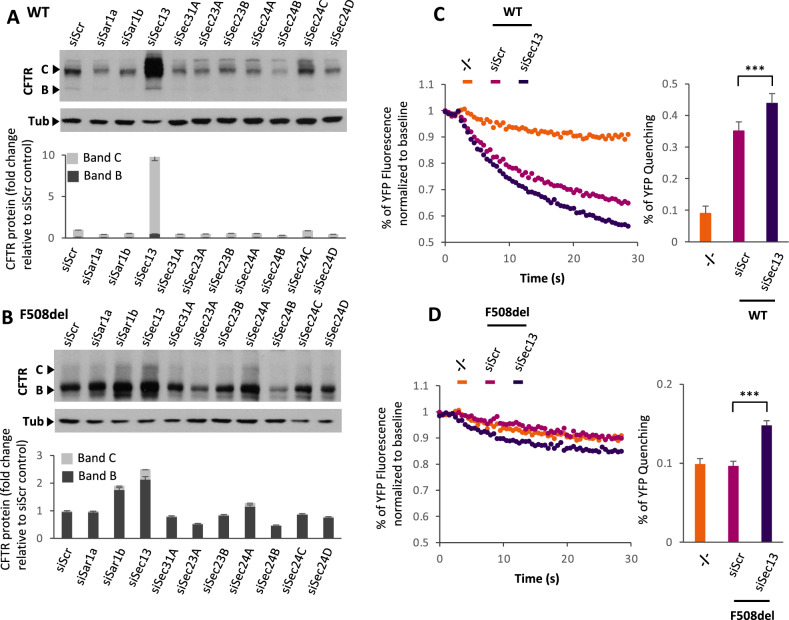
siSec13 increases CFTR export and function. (**A**, **C**) Immunoblot analysis (upper) and quantifications (lower) of CFTR expression following transfection of HBE41o- (WT CFTR) (**A**) and CFBE41o- (F508del CFTR) (**C**) cells with the independent siRNA targeting the different COPII core subunits. Data are presented in lower panel as fold-change of CFTR band B and band C relative to siScr control transfection (mean ± SEM, n ≥ 3). (**B**, **D**) Representative fluorescent imaging plate reader traces (left) and quantification (right) of YFP-quenching following transfection of HBE41o- (WT CFTR) (**B**) and CFBE410- (F508del) (**D**) YFP cells with either siSec13 or siScr control compared to the -/- YFP cells not expressing CFTR. Data are presented as % relative to baseline (mean ± SEM, n ≥ 3). In all panels, ∗ indicates significant differences (p < 0.05) relative to siScr transfection determined by two-tailed t-test. See Fig. S9 for non-cropped blots used to generate figure.

To begin to address the apparent complex role(s) of Sec13 in facilitating endomembrane biology, we first focused on its known role in ER export by quantifying the effect of siSec13 on the expression level of the COPII components using immunoblotting (Fig. 2D). We noted a modest increase of Sec24A and a small decrease of Sec24C, but a drastic decrease of Sec31A, a result consistent with the proteomic datasets (Figs. 2A,  3A, B) and, as indicated above, the dependance of Sec31A on Sec13 for its stability^32,114,115^ (Fig. 2D). Strikingly, within 24 h we saw a strong (ten-fold) stabilization of the mature band C glycoform of WT CFTR in response to Sec13 reduction reflecting successful export from the ER and processing by Golgi-associated glycosylation enzymes (Fig. 2D). This response was similar to the strong increase in the level of the mature ABCC4 glycoform, also known as multidrug resistance protein 4 (MDRP4)^116^, a protein similar to CFTR as part of the ABC transporter family (ABCC7)^117^. We also determined the level of select proteins localized in different compartments of the cell or extracellular space following secretion (Fig. 2E). For example, we observed a significant decrease in the level of TGN38 impacting post-trans Golgi network function and in maintaining TGN morphology^118,119^. Interestingly, we observed a drastic diminution of Niemann-Pick disease Type C1 (NPC1), a protein that is a resident protein in the lysosome (Fig. 2E), suggesting an important role of Sec13 cage assembly in managing resident cargo flow to the lysosomal network to prevent degradation in the ER^13,21,23,31,39,112,120^ and/or, for example, the general stability of the lysosomal system in response to the dependence of GATOR2 complex function on Sec13^65–67^.

To determine if Sec13 silencing affected WT CFTR degradation in lysosomes, which could be either due to trafficking directly from ER or during recycling between the cell surface through the endosomal-lysosomal system, we treated WT expressing HBE41o- cells with the lysosome-autophagosome inhibitor chloroquine^121^. In the absence of siSec13 silencing we observed a strong stabilization of CFTR band C glycoform to chloroquine which remained unaltered in response to siSec13 silencing (Fig. 2G). These results suggest that the post-ER cell surface recycling pool degraded by the lysosome confers increased stability to mature CFTR during the time-frame of siSec13 treatment (Fig. 2G). In contrast, we noted only a minor change in the ER band B CFTR glycoform (Fig. 2D), indicating that Sec13 silencing had little to no effect on degradation of ER residual glycoforms normally processed through ER-associated degradation (ERAD)^20,68,88,90,122–129^. These observations are consistent with a previous report showing that Sec13 silencing leads to a strong defect of autophagy in *Saccharomyces cerevisiae*^130^, and consistent with the observation that autophagy modulation can partially correct F508del CFTR stability and trafficking^82^. Interestingly, we saw no effect on the total secreted pool of the ER-generated extracellular protease alpha-1-antitrypsin (AAT). These results raise the possibility that the recycling pool of its abundant ER export receptors LMAN1^46^ and/or SURF4^46,131–133^ may be insensitive to the time-frame (24 h) of siSec13 silencing used to follow trafficking of CFTR and ABCC4 that utilize unique ER exit motifs for direct delivery to cell surface^34,41,70^. Likewise, siRNA Sec13 had no effect on the transferrin-receptor (TFRC)^134,135^ protein level in the time frame (24 h) used to detect increased WT CFTR band C stability, suggesting that Sec13 has no or minimal role in managing the long-term stability of late endocytic recycling pathway components (Fig. 2H).

### The effect of Sec13 silencing on variant F508del CFTR export and function

To address how the COPII system through Sec 13 can discriminate between differentially folded states of protein cargo in vivo, we dissected the functional contribution of each of the COPII components for variant CFTR proteins. There are over 2000 variants of CFTR in the CF population that differentially contribute to disease onset. An analysis of the allele frequency of CF-causing mutations reveals that approximately 70–80% of patients carry at least one copy of a three base pair deletion leading to the loss of a phenylalanine at position 508 (F508del) in NBD1^136–139^. The F508del mutation disrupts the folding of the variant protein, leading to its loss of engagement by COPII in the ER compartment and clearance by ER-associated degradation (ERAD)^76,77,140–144^. Using Gaussian process (GP)-based machine learning with 71 variants triggering CF in the population^4^, we recently demonstrated^1–7,145^ the existence of a thermodynamically sensitive region of the CFTR fold involving the interfaces between nucleotide binding domain 1 (NBD1) contributed by the F508 and R560 residues, and the fourth intracellular loop 4 (ICL4) of transmembrane (TM) domain 2 (TMD2), the latter being responsible for COPII engagement for trafficking from the ER via the di-acidic exit motif^3,4^. We have shown that loss of function of the di-acidic code in the NBD1 domain is the primary defect responsible for CF in F508del-expressing patients^3,4,7,41,50,70^. This observation is consistent with MS analysis of residues undergoing methylation and ubiquitination where ubiquitination of di-acidic motif residues targets CFTR for degradation when challenged through misfolding by the F508del allele change^87,146^, and consistent with the need to assemble the ICL4 and NBD1 domains to achieve export^73–75,77,85,86^.

Using SDS-PAGE followed by immunoblotting to capture the conversion of the ER-localized band B glycoform to the Golgi-associated band C glycoform in response to export (Fig. 3A, B (% band B)), HBE41o- cells stably expressing WT CFTR (Fig. 3A) and CFBE41o- stably expressing F508del CFTR (Fig. 3B) were transfected with siRNA specific for each of the COPII components (Fig. 3A, B; Supplementary Fig. S5, WT CFTR; Supplementary S5, F508del CFTR). The level of silencing of each component was validated by immunoblotting (Supplementary Fig. S5). While we observed that silencing of various COPII components differentially modulate WT and F508del CFTR stabilization and trafficking (Fig. 3, Supplementary Fig. S5), only silencing of Sec13 yielded a strikingly strong (~ ten-fold) increase in stabilization of WT CFTR band C glycoform (Fig. 3A). Correspondingly, it also yielded a nearly 2.5-fold increase in total F508del band B glycoform (Fig. 3B). The next highest effect, but only for F508del CFTR, was silencing of Sar1b, potentially reflecting the increased sensitivity of general COPII assembly for recruitment of folding defective proteins in response to decreased Sec13/31 cage assembly complex (Fig. 3A, B; Supplementary Fig. S5). Specificity of Sec13 silencing was validated by using an siRNA recognizing a different region of Sec13 coding sequence (Supplementary Fig. S6). Notably, whereas WT-CFTR accumulated in the band C post-ER glycoform in response to siSec13, F508del accumulated largely as the band B glycoform with a modest but significant increase in band C (Fig. 3B, upper panel, siSec13 lane (band C arrow); quantitation lower panel). These results indicate that siSec13 is not rescuing F508del misfolding for export as do proteostasis components^68,82,88–90^ and CFTR modulators^3,4,7,42,125,126,147–152^.

To determine whether changes in steady state levels of Sec13 are responsible for protein stability and/or trafficking of WT CFTR, we performed a time-course of Sec13 silencing for 2, 3 and 4 days. We observed that decreasing levels of Sec13 leads to a correspondingly increase in WT CFTR band C that peaked by day 3 (Supplementary Fig. S6). To determine if the siSec13 stabilization and trafficking of CFTR was dependent on COPII assembly through the cargo collecting components Sec23/24, we knocked-down all Sec24 isoforms, thereby preventing CFTR WT or F508del recognition by the COPII system (Supplementary Fig. S6). We observed that the deletion of Sec24 isoforms eliminated siSec13 enhancement of CFTR stabilization and export for both WT and F508del, suggesting that the CFTR stability following Sec13 silencing requires the full functionality of the COPII machinery (Supplementary Fig. S6). The combined results suggest that reduction of the cage assembly complex Sec13/31 in response to decreasing levels Sec13 during silencing improves stability of WT CFTR pool in the ER for export, but only weakly corrects the ability of misfolded F508del for export.

We next determined if the partial rescue of band B F508del and the substantial increased band C fraction of WT CFTR represented a functional cell surface-localized chloride channel. In order to assess the functional status of WT and F508del CFTR treated with siSec13, we used HBE41o- and CFBE41o- cells stably expressing the halide-sensitive YFP-H148Q/I152L, a yellow fluorescent protein (YFP) variant whose fluorescence can be quenched in response to iodide influx entering the cell through a functional, cell surface localized CFTR channel^153^. When compared to cells lacking CFTR where quenching is not observed (Fig. 3C, orange), we observed that Sec13 silencing led to an increase in function at the cell surface for both WT (Fig. 3C) and F508del (Fig. 3D) consistent with its improved stability.

Combined, these results suggest that the role of Sec13 cage assembly is distinct from features such as the Sec23/24 cargo selection machinery dictating initial recruitment of cargo, but is consistent with a role in improving the overall availability of CFTR for ER export by reducing degradation at the ERES-ERGIC interface where COPII-COPI exchange differentiates export from recycling for degradation^34,49,51,57,82,89,91,154–156^, or in the case of WT CFTR, post-ER recycling to the lysosome sensitive to Sec13’s role in maintaining the lysosome. These results surprisingly suggest that steady-state levels of the Sec13 affecting the generation of the Sec13/31 complex regulates CFTR stability in both healthy and misfolded states and the more global state of the function of the early endomembrane system.

### Sec13 silencing decreases CFTR ubiquitination and degradation via ERAD

To address the mechanism of action of Sec13 management of CFTR stability, chaperone assisted protein folding through proteostasis and ERAD have been shown to play an important role in triaging proteins for export by the COPII export machinery^62,76,82,123,157^. Newly synthesized proteins that do not fold correctly in the ER are generally targeted to the ubiquitin–proteasome ERAD pathway associated with retrograde trafficking from ERES and ERGIC^20,51,57,62,158^ facilitated by COPII-COPI exchange^34,49,51,57,82,89,91,154–156^. In addition, there is now recognized a COPII dependent autophagy pathway that delivers ER cargo directly to the lysosome^13,21,23,31,39,112,120^. One unusual feature of CFTR protein biogenesis is that during translation in heterologous expression systems, and as observed in primary cells expressing endogenous levels of CFTR, approximately 75% of WT CFTR and most variant F508del CFTR protein is thought to be degraded by the ERAD pathway- suggesting the existence of metastable state in fold design that is not responsive to the steady-state ER associated proteostasis machinery facilitating normal folding in multiple cell types^3,4,49,77,78,82,89,90,159,160^.

**Figure 4:**
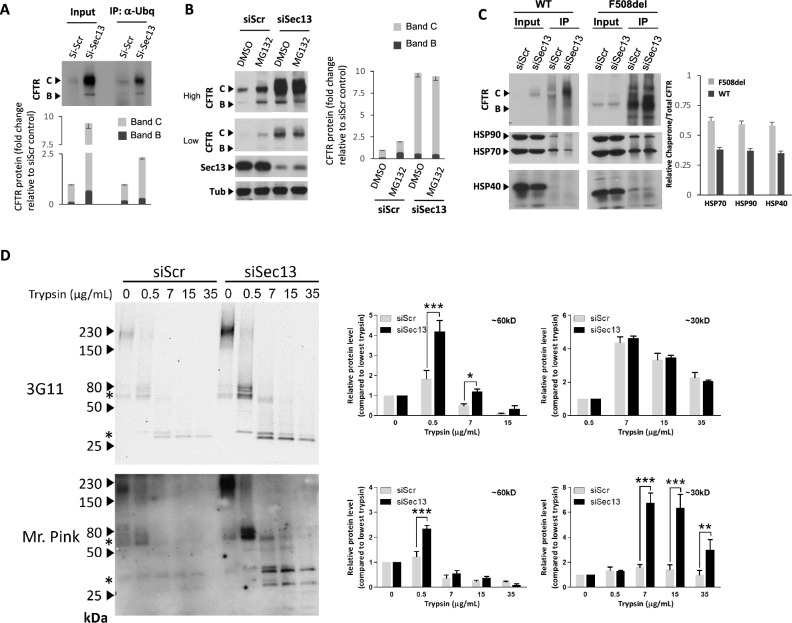
siSec13 decreases CFTR ubiquitination, degradation via ERAD and increase CFTR stability. (**A**) Immunoblot (upper) and quantification (Lower) of CFTR expression in ubiquitin-containing immunoprecipitates following siSec13 transfection in HBE410- cells compared to siScr. Data are presented in lower panel as fold-change of CFTR band B and band C relative to siScr control transfection (mean ± SEM, n ≥ 2). (**B**) Immunoblot (left) and quantification (right) of CFTR expression (high and low exposure) following transfection of HBE41o- cells with siSec13 in combination with MG132 treatment compared to DMSO and siScr controls. Data are presented in the right panel as fold-change relative of CFTR band B and band C to siScr control transfection in combination with DMSO treatment (mean ± SEM, n ≥ 3). (**C**) Immunoblot (left) and quantification analysis (right) for CFTR, Hsp90, Hsc/p70 and Hsp40 in CFTR-containing immunoprecipitates following siSec13 transfection of HBE410- (WT) and CFBE41o- (F508del) cells compared to siScr (set to a value of 1, y-axis) (mean ± SEM, n ≥ 2). (**D**) (Upper panels) Representative immunoblot (left panel) from two experimental replicates of HBE41o- before and after proteolysis digestion with increasing concentration of trypsin (mg/ml) showing digestion pattern of the 60 kDa NBD1 domain and its protease resistant 30 kDa fragment (middle and right panels, respectively) using 3G11 antibody Two-Way ANOVA was applied to compare protein levels between siRNA Sec13 and controls (***, p < 0.001; **, 0.001 < p < 0.01; *, 0.01p < 0.05). (Lower panels) 60 and 30 kDa trypsin fragments and their quantitation recognized by a polyclonal antibody (Mr. Pink) that recognizes multiple CFTR epitopes^75,85,86,171^. See Fig. S10 for non-cropped blots used to generate figure.

To understand the stabilization of WT CFTR upon siSec13 treatment, we first investigated the ubiquitination status of CFTR after silencing Sec13 by immunoprecipitation (Fig. 4A, Fig. S10B). siSec13 treated HBE41o- (WT) cell lysates were incubated with ubiquitin antibody and eluates were separated on SDS-PAGE gels under denaturing conditions (Fig. 4A). We observed that Sec13 silencing promoted a significant reduction of WT CFTR band B and band C levels recovered in ubiquitin immunoprecipitates (Fig. 4A), indicating that abrogation of Sec13 decreased the ubiquitination of WT CFTR, limiting its degradation leading to its increased stabilization. In addition, we investigated the possible additive and/or synergistic effect of siSec13 treatment with the proteasome inhibitor compound MG132 known to increase WT CFTR stability^161^ (Fig. 4B). Here, and as expected, we observed a strong MG132 effect on increasing WT CFTR stability and CFTR trafficking in HBE41o- (WT) lung cell lines (Fig. 4B). Interestingly, addition of MG132 to siSec13 treated cells did not result in any additive or synergistic effect between the two treatments alone (Fig. 4B). Given the observation that the impact of siSec13 on WT CFTR was much higher than the impact observed after MG132 treatment, these results suggest siSec13 modulates additional pathways managing CFTR stability- perhaps reflecting the role of the Sec13/31 complex in ER function at the ERES-ERGIC interface involved in COPII-COPI exchange^9,51,57,77,78,89,90,155,156^, and/or in its involvement ER-autophagy pathways.

One possibility for enhanced stability of CFTR is that Sec13 silencing induces ER stress leading to alteration of chaperone levels supporting ER function. We followed the siSec13 response levels of calnexin (a resident luminal chaperone), luminal Grp94 (an Hsp90 paralog) and ribophorin B (a resident membrane protein associated with the ER) (Supplementary Fig. S7) with Hsp90 serving as a cytosolic reporter. We did not observe any modification of the level of these proteins upon Sec13 silencing suggesting that siSec13 does not impact the general ER proteostasis environment through stress related unfolded protein response (UPR) pathways in the short experimental timeframe (24 h) impacting CFTR stability (Fig. 3, Supplementary Fig. S7). The possibility of a change in CFTR mRNA levels upon Sec13 silencing was examined by quantitative RT-qPCR (Supplementary Fig. S7B). There was no difference in CFTR mRNA levels in the Sec13 depleted samples compared to controls, suggesting that the upstream roles of Sec13 affecting chromatin architecture and transcription potentially impacting the level of the Sec13 transcript do not play a role in CFTR stabilization in the limited (24 h) time-frame of experiments.

### Sec13 promotes CFTR rescue through managing its response to chaperones

Given the extensive cytoplasmic orientation of its non-transmembrane domains, CFTR normally interacts with the chaperone network found in the cytoplasm^76,77,82,142,161,162^. We have previously shown that chronic activation of the cytosolic heat shock response (HSR) in F508del expressing cells is counterproductive—referred to as the maladaptive stress response (MSR)^161^. In the augmented proteostasis environment in response to misfolding of F508del, the variant is retained in the ER by an increase in Hsp70 and Hsp90 chaperone pools forming a ‘chaperone trap’ that we have posited is attempting to resolve unwanted structural intermediates that are a challenge to the cytosolic folding machinery rendering it susceptible to degradation by ERAD^89^.

To address the potential role of cytosolic chaperones in response to Sec13 silencing, we compared the chaperone pools associated with either WT or F508del CFTR in siRNA control or siRNA Sec13 treated cells. CFTR was immunoprecipitated from control or Sec13 depleted HBE41o- and CFBE41o- cells, respectively, and the bound chaperone pools were analyzed using chaperone specific antibodies to the cytosolic Hsp40, Hsp70 and Hsp90^82^. As expected^69,89^, analysis of CFTR bound chaperone proteins in the control revealed that mutant F508del protein shows a stronger association with chaperones in comparison to WT CFTR (Fig. 4C). Upon Sec13 silencing, quantification of the bound chaperone proteins in the immunoblots relative to that of siScr control (set to a value of 1) showed a decreased association of Hsp40, Hsp70 and Hsp90 to both WT- and F508del, indicating that reduction of Sec13 levels decreases the interactions between CFTR and proteostasis components directing the maladaptive chaperone response^161^, and therefore limits interaction of CFTR in the ER with the chaperone trap^89,161^. Loss of this interaction impacts COPII/COPI exchange for targeting to degradation at the ERES-ERGIC interface^9,20,51,57,77,78,89,90,155,156,161^.

### Protease digestion reveals increased CFTR fold stability in response to siSec13

Since Sec13 silencing reduced the targeting of both WT and F508del CFTR for degradation, we assessed the impact of Sec13 silencing on improving CFTR conformational stability. We followed CFTR stability using protease digestion^74,75,77^ to detect the conformation status of CFTR protein in response to reduced Sec13 proteins levels. Conformationally stable proteins generally have more compact three-dimensional structures than their variant counterparts, leading to enhanced resistance towards protease digestion. Using the NBD1 60 kDa domain specific antibody 3G11 (Fig. 4D, upper panels) and a polyclonal antibody (Mr. Pink) (Fig. 4D**,** lower panels)^73–75,83,84,163,164^, we observed the appearance of a more slowly migrating trypsin resistant bands in Sec13 depleted samples compared to siScr control, indicating an increase in conformation stability (Fig. 4D). For example, we observed the appearance of an increased level of a protease resistant band of 60 kDa, characteristic of a stabilized NBD1 domain, and 30 kDa stable region (Fig. 4D, left panel immunoblots, middle panel 60 kDa band quantitation, right panel 30 kDa band quantitation).These results suggests that siSec13 treatment leads to enhanced protease protection of more folded state of NBD1 (Fig. 4D) that, while insufficient to rescue the key YKDAD di-acidic code exit motif defective in the F508del variant for export to the surface^3,4,7^, helps to prevent delivery of CFTR to ERAD and/or COPII sensitive autophagic pathways at the COPII/COPI ERES-ERGIC interface^122^.

## Discussion

Using CFTR as a model COPII dependent cargo we have defined the role of Sec13 to determine where in the hierarchy of its interactions it exerted its strongest and more immediate effects to understand its mechanism(s) of action in the cell. We screened a library of siRNAs silencing of each of COPII subunits and their isoforms to only reduce their expression. Complete silencing of Sec13 would likely be lethal to the cell as it would eliminate ER export for all proteins, consistent with results in yeast^114,115,165^. Surprisingly, we found that by reducing Sec13 protein levels, unlike any of the other COPII components such as Sec23/24 involved in cargo selection, we significantly improved both WT and F508del CFTR stability by reducing its degradation. Consistent with this conclusion, partial Sec13 silencing also resulted in a decrease in HSPs bound to CFTR, helping to avoid the chaperone trap^89^. Whereas WT CFTR showed improved accumulation as the cell surface band C glycoform, F508del largely accumulated as an ER localized band B in response to reduced levels of Sec13/31 cage assembly complexes, although we did detect a modest increase of band C and improved cell surface channel activity, reflecting the stabilizing and trafficking effect observed for WT CFTR. The stability increase in WT and F508del variant CFTR could reflect a contribution of both prevention of delivery to ERAD and/or autophagic/lysosomal including the mTOR pathways and potentially linked to nutrient responses^166^ managed by Sec13 at the ERES-ERGIC interface responsible for COPII-COPI exchange^20,48,51,57,91,155^. Moreover, it remains possible that non-conventional trafficking pathways from the ER could also be susceptible to increased activity in response to reduction of Sec13 levels^16,166,167^.

Intriguingly, the reduced level of Sec13 still allowed for delivery of WT CFTR to the cell surface demonstrating that the cargo selection step for export through the di-acidic code was complete and flow to cell surface is unimpeded following partial Sec13 silencing, suggesting that WT CFTR escapes the COPI dependent recycling step for degradation. Moreover, Sec13 sensitive post-Golgi endo-lysosome trafficking pathways sensitive to Sec13 function may also contribute to stability reflecting its more global role(s) in management of endomembrane composition. A similar result was observed for the ABC transporter family member ABCC4. These results are consistent with improved stability of the fold in response to protease digestion, suggesting that CFTR matures past early intermediate metastable forms in the presence of reduced Sec13/31 complex.

**Figure 5:**
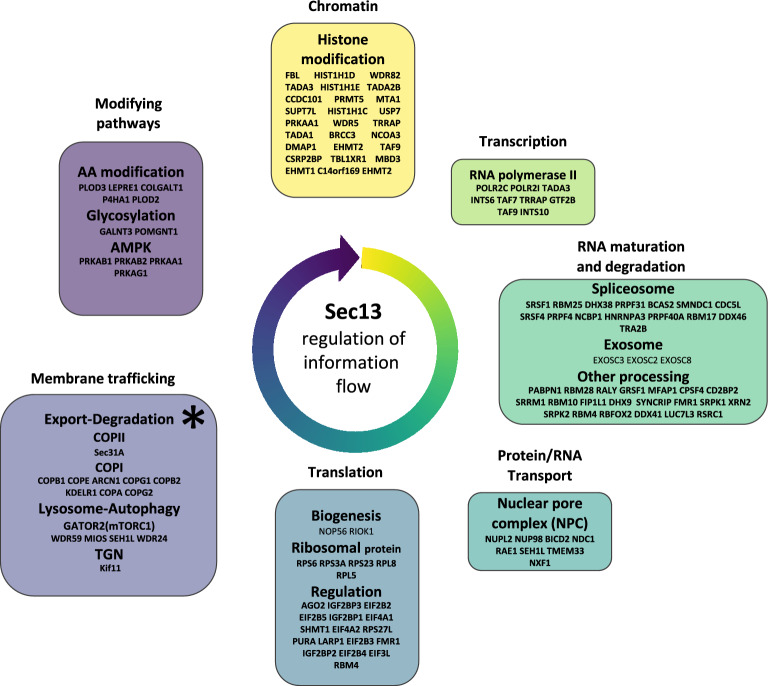
Sec13 is a central regulator of early information flow in eukaryotic cells. Sec13 participates in a wide range of cellular functions related to initiation of information flow from the genome through the endomembrane pathway starting with chromatin condensation/de-condensation, modulation of transcription, RNA maturation and degradation, RNA/protein transport between nucleus and cytoplasm, translation of protein, protein trafficking and modulation of key signaling pathways affecting post-ER endomembrane pathways. Proteins associated with these functions were recovered in the Sec13 interactome. The combined results suggest that Sec13 provides pivotal framework around which endomembrane trafficking could be coordinated through a SCV set-point^6,7^ defined by Sec13 activity in each cell type in generating and mobilizing cargo that contributes as a collective to cellular endomembrane design and function as a quality system^3,4^.

We now suggest that Sec13/31 cage assembly, globally managed by Sec13 levels in the cell, impacts the dynamics between COPII forward and COPI reverse recycling pathways at the ERES-ERGIC interface as originally suggested^57^ and later supported the efforts of many others^20,48,51,57,91,155^. The effect on different cargos will require further investigation given that neither AAT, a secreted protein, nor TRF, a plasma membrane recycling protein, were impacted by siRNA Sec13 in the time-frame used to capture impact on nascent synthesis of CFTR. These results potentially reflect the role of recycling cargo receptors such as LMAN1 and SURF4 for AAT^18,46^, or endosomal recycling pathways for TRF^134^. In addition, reduction of Sec13 may influence the activity of bypass pathways that we and others have reported^51,166^. We conclude an important role for Sec13 is in establishing a ‘set-point’ for differentiation between export and degradation with reduced levels of cage assembly favoring cargo stability associated with improved export through intrinsic export motifs such as the di-acidic code for CFTR (Fig. 5, asterisk).

While we focused on the role of Sec13 in COPII mediation of cargo traffic from the ER, from a more global perspective based on our Sec13 interactome hits and the response of the cell proteome to siSec13 based silencing, we now appreciate that Sec13 is involved in a large number of activities that could also influence the organization of information flow initiated by expression from the genome (Fig. 5, see summary Supplementary Fig. S1). While we did not detect a change in Sec13 mRNA levels during the short 24 h window leading to the improved stability of CFTR, the more global changes in Sec13’s physical association with chromatin, gene transcription factors affecting RNA maturation, RNA/protein transport from the nucleus to the cytoplasm, as well as translational and trafficking components, in response to Sec13 silencing highlights its potential for a much broader role in coordinating endomembrane structure to function relationships. One possibility is that Sec13’s WD40 propeller domain, a domain that is known to function in generating protein–protein interactions, plays a key role in coordinating information flow by as yet unanticipated mechanisms, to build as a collective endomembrane systems that are dependent on the genome encoded specialized cargo composition of different cell types^49^. This conclusion is consistent with our recent results where we have used a Gaussian process (GP) machine learning framework to describe the spatial covariance (SCV) features dictating sequence-to-function-to-structure relationships^4^ responsible for endomembrane trafficking of CFTR^3,4,7^ and other cargo^1,5,6,145^ on a residue-by-residue basis as an integrated quality system (QS) in each cell type in response to inherited genetic variation triggering human disease^3,4^. By being positioned at the root of the genome encoded exocytic and endocytic endomembrane system that are different for each cell type and highly responsive to their local environments, we suggest that Sec13 serves as an unanticipated key and dynamic regulatory node to achieve globally the genome to proteome transformation responsible for health and disease. Such flexibility at the first step in the exocytic pathway allows each cell type to (re)program SCV relationships in response to variation across the population to provide a precision response to natural selection responsible for survival and fitness^1–7^ (Fig. 5).

## Materials and methods

### Cell culture, transfection, and treatment with compounds

#### Cell culture

HBE41o- and CFBE41o- expressing WT and F508del CFTR, respectively, were obtained from Dr. J Clancy (University of Alabama). Cells were cultured in α-MEM containing 10% fetal bovine serum (FBS) and 2 mM l-glutamine. HBE41o- and CFBE41o- containing YFP-H148Q were engineered and cultured as previously described^168^. *siRNA-mediated silencing.* HBE41o- and CFBE41o- cells were plated in 12-well dishes and grown to 60–70% confluence. COPII subunit silencing was performed using 50 nm of validated siRNA for the indicated COPII subunit as per manufacturer’s directions. Cells were cultured for 5 h in the serum-free opti-MEM containing transfection complexes and subsequently washed and cultured for an addition of 48 h in the presence of growth medium prior to processing for immunoblot analysis. *Treatment.* Where indicated, cells were treated with MG132 (10 µM), chloroquine (50 µM) in complete growth media and incubated at 37 °C, 5% CO2 for 24 h. MG132 and chloroquine were procured from MedChemExpress.

### Immunoblot and immunoprecipitation

#### Preparation of cell lysate and immunoblotting

As previously described^3,4^, cells were washed twice with ice-cold PBS, and 50 µl of lysis buffer (50 mM Tris–HCl, pH 7.4, 150 mM NaCl, 1% Triton X-100, and 2 mg/ml of complete protease inhibitor mixture) was added to each well. Cells were lysed on ice for 30 min with occasional mixing. The lysates were collected and spun at 14,000 × g for 20 min at 4 °C. The protein concentration in the supernatants was assessed by Bradford assay using the Coomassie Protein Assay Reagent (Pierce). 15 µg of total protein were separated by 7% SDS-PAGE, transferred to nitrocellulose, and incubated with specific primary antibody (Abcam) over-night at 4 °C in TBS + 0.1% Tween 20 + 5% milk. Nitrocellulose was subsequently incubated with the appropriate secondary antibody (Abcam) in TBS + 0.1%Tween 20 + 5% milk at room temperature for 1 h. Detection was performed by chemiluminescence using ECL reagent. *Immunoprecipitation.* Lysates were prepared as described for immunoblotting. 1.0 mg of total protein was pre-cleared by incubating with γ-bind beads (GE Healthcare) for 1 h at 4 °C and subsequently incubated for 16 h at 4 °C with 20 µl of either CFTR, ubiquitin or Sec13 antibody cross-linked to γ-bind beads (GE Healthcare). The monoclonal antibody to Sec13 was generated and characterized using purified Sec13 as described previously^99^. The beads were washed with two changes of lysis buffer without protease inhibitor mixture. Beads were pelleted at 2000 × g for 1 min, and bound proteins were eluted by incubation with 50 mM Tris–HCl, pH 6.8, containing 1% SDS at rt for 30 min and at 37 °C for 5 min. Immunoblots of eluted proteins were analyzed as described above. The immunoblots were developed using x-ray film- therefore, the markers do not appear on the blots. The membranes were cut (using Ponceau for protein visualization) before probing with primary antibodies allowing us to probe different regions of the same membrane for different proteins.

### CFTR trypsin proteolysis

CFTR trypsin digestion has been performed as previously described^161^. Briefly, HBE41o- cells treated with siSec13 or control siScr, were first lysed for 30 min at 4 °C with lysis buffer (50 mM Tris–HCl pH 7.4, 150 mM NaCl, 1% Triton X-100,2 mg/ml Protease Inhibitor cocktail [Roche]) and harvested at 20,000 g for 20 min at 4 °C. Total protein concentration of pre-cleared lysates was determined by Bradford. Proteolysis was performed by incubating 80 mg of total protein with increasing concentration of Trypsin in PBS (0.05–0.35 mg/ml) at 4 °C for15 min. Proteolysis was stopped by adding 1 mM of PMSF and 6 × SDS-PAGE sample buffer. Samples were loaded onto 12% SDS-PAGE for separation of the proteolytic fragments and probed with CFTR antibodies for NBD1 (3G11: epitope 396–405).

### q-RT-PCR

Quantitative Reverse Transcription PCR (qRT-PCR) was performed using the iScript One-Step RT-PCR kit with SYBR green (BioRad, Hercules, CA). RNA was standardized by quantification of beta-glucuronidase (GUS) mRNA, and all values were expressed relative to GUS. CFTR Forward (GTGGCTGCTTCTTTG-GTTGT) and reverse (CGAACTGCTGCTGGTGATAA) primers were used as indicated.

### Functional YFP quenching assay

As described previously^3^, YFP expressing F508del-CFBE41o- and WT-CFBE41o- cells stably transfected with pcDNA3.1 containing YFP-H148Q were provided by Dr. L. Galietta, Telethon University for Genetics and Medicine, Puzzuoli, IT. The stable cell lines could be passed at least 15 times without a decrease in YFP-H148Q fluorescence. F508del-CFBE -YFP and WT-CFBE-YFP were cultured in the same media as the YFP non-expressing cells with addition 0.75 mg/ml G418. Cells were treated with indicated drug and siRNA. Following treatment, cells were stimulated with a final concentration of 10 μM Fsk and 50 μM Gen for 15 min prior to addition of PBS + NaI (replacement of NaCl with 137 mm NaI). Fluorescence was monitored each second for a total of 30 s (3 s prior to addition of NaI and 27 s after addition of NaI) using a Synergy H1 HybridReader (BioTek, Winooski, VT). As a negative control, identical experiments were performed with null -/- HBE41o- -YFP cells not expressing F508del CFTR.

### Immunoprecipitation and sample preparation for Sec13 interactome

HBE41o- cell lysates were prepared as described for immunoblotting^89,108,146^. The lysates were pre-cleared using 50 μl of GammaBind Plus Sepharose beads for 1 h at 4 °C with mixing. The beads were pelleted at 500 × g for 5 min at 4 °C and the pre-cleared lysate was transferred to a new tube. The Sec13 immunoprecipitation was performed by adding the Sec13 antibody pre-crosslinked to GammaBind Plus Sepharose beads to the precleared lysates (4 mg total protein per IP). Control IPs were performed by using mock-IPs, in which no antibody is coupled to the beads, to identify bead- and cell-specific background. The lysates were incubated with or without the antibody overnight at 4 °C with end-over-end mixing. The beads were pelleted at 500 × g for 5 min at 4 °C and washed twice with 10 bead volumes of lysis buffer and twice with 10 bead volumes of lysis buffer without IGEPAL-CA630. Following immunoprecipitation washes with lysis buffer and 50 mM ammonium bicarbonate, proteins were digested directly on-beads as previously described^169^. Briefly, proteins bound to the beads were resuspended with 8 M urea and 50 mM ammonium bicarbonate, and Cys disulfide bonds were reduced with 10 mM Tris (2-carboxyethyl) phosphine at 30 °C for 60 min followed by cysteine alkylation with 30 mM iodoacetamide in the dark at room temperature for 30 min. Following alkylation, urea was diluted to 1 M urea using 50 mM ammonium bicarbonate, and proteins were finally subjected to overnight digestion with mass spec grade Trypsin/Lys-C mix (Promega, Madison, WI). Finally, beads were pulled down and the solution with peptides collected into a new tube. The beads were then washed once with 50 mM ammonium bicarbonate to increase peptide recovery. The digested samples were desalted using a C18 TopTip (PolyLC, Columbia, MD), and the organic solvent was removed in a SpeedVac concentrator prior to LC–MS/MS analysis.

### Sample preparation and TMT labelling for proteome profiling

Protein extracts were obtained from HBE41o- cells treated with siSec13 or siScr (control). Cell lysis was performed as described above. TMT labelling was prepared as previously described^89,108,146^. 50 µg of a protein aliquot from each sample was acetone precipitated. The protein pellets were resolubilized in 100 mM TEAB supplemented with 0.1% SDS, reduced with TCEP for 1 h at 55 °C, and then alkylated with iodoacetamide for 30 min in the dark. The denatured proteins were digested with trypsin by overnight incubation at 37 °C. Resulting peptides were labelled with 6-plex Tandem Mass Tag (TMT) labelling reagent (Thermo-Fisher) according to the manufacturer’s recommendations. Subsequently, insoluble precipitates were removed by centrifugation (15 min, 18,000 g) and samples reduced to near dryness *in vacuo*.

### 2D LC MS/MS analysis

2D LC MS/MS analysis was performed as previously described^89,108,146^. Briefly, dried samples were reconstituted in 100 mM ammonium formate (pH ~ 10) and analyzed by 2D LC–MS/MS using a 2D nanoACQUITY Ultra Performance Liquid Chromatography system (Waters Corp., Milford, MA) coupled to a Q-Exactive Plus mass spectrometer (Thermo Fisher Scientific). Peptides were loaded onto the first-dimension column, XBridge BEH130 C18 NanoEase (300 μm × 50 mm, 5 μm) equilibrated with solvent A [20 mM ammonium formate (pH 10), first dimension pump] at 2 μl/min. The first fraction was eluted from the first-dimension column at 17% of solvent B (100% acetonitrile) for 4 min and transferred to the second dimension Symmetry C18 trap column 0.180 × 20 mm (Waters Corp., Milford, MA) using a 1:10 dilution with 99.9% second dimensional pump solvent A (0.1% formic acid in water) at 20 μl/min. Peptides were then eluted from the trap column and resolved on the analytical C18 BEH130 PicoChip column 0.075 × 100-mm, 1.7-μm particles (NewObjective, MA) at low pH by increasing the composition of solvent B (100% acetonitrile) from 2 to 26% over 94 min at 400 nl/min. Subsequent fractions were carried with increasing concentrations of solvent B. The following 4 first dimension fractions were eluted at 19.5%, 22%, 26% and 65% solvent B. The mass spectrometer was operated in positive data-dependent acquisition mode. MS1 spectra were measured with a resolution of 70,000, an AGC target of 1,000,000, and a mass range from 350 to 1700 m/z. Up to 12 MS2 spectra per duty cycle were triggered, fragmented by HCD, and acquired with a resolution of 17,500 and an AGC target of 5e4, an isolation window of 2.0 m/z and a normalized collision energy of 25. Dynamic exclusion was enabled with duration of 20 s.

### Data analysis

As previously describe^89,108,146^, mass spectra were analyzed with MaxQuant software version 1.5.5.1. MS/MS spectra were searched against the Homo sapiens Uniprot protein sequence database (version July 2016) and GPM cRAP sequences (commonly known protein contaminants). Precursor mass tolerance was set to 20 and 4.5 ppm for the first search where initial mass recalibration was completed and for the main search, respectively. Product ions were searched with a mass tolerance 0.5 Da. The maximum precursor ion charge state used for searching was 7. Carbamidomethylation of cysteines was searched as a fixed modification, while oxidation of methionines and acetylation of protein N-terminal were searched as variable modifications. Enzyme was set to trypsin in a specific mode and a maximum of two missed cleavages were allowed for searching. The target-decoy-based false discovery rate filter for spectrum and protein identification was set to 1%. As previously described^170^, normalized spectral abundance factor (NSAF) values were calculated for proteins in each sample to account for protein size and variability between runs. The NSAF for a protein p is the number of spectral counts (SpC, the total number of MS/MS spectra) identifying a protein, p, divided by the protein length (L), divided by the sum of SpC/L for all N proteins in the experimental design. The data sets were statistically compared to determine the significance of the change between the two groups using Student’s t test (two tailed unpaired t test). To determine the relative abundance of interacting proteins on Sec13 cross-linked Gamma Bind Plus Sepharose beads relative to the Gamma Bind Plus Sepharose beads alone, the data sets were first filtered to include only those proteins that were detected in all three replicates for each condition. Then the ratio of the mean of the NSAF values from three biological replicates of Sec13 immunoprecipitates to the mean of NSAF values from three biological replicates of the control was computed to calculate the fold-change.

### Supplementary Information

Supplementary Tables.

Supplementary Information 2.

Supplementary Figures.

## Data Availability

All data generated or analyzed during this study are included in the published article and its supplementary information file.
