# Supplementary material for: COPII cage assembly factor Sec13 integrates information flow regulating endomembrane function in response to human variation
Source: Sci Rep. 2024 May 3;14:10160. doi: 10.1038/s41598-024-60687-2 (PMC11065896; doi:10.1038/s41598-024-60687-2)
Supplement: Supplementary file 2 — Supplementary Information 2. [file 41598_2024_60687_MOESM2_ESM.docx]

**Supplemental Figure Legends**

**Figure S1. Sec13 interactome supplementary data.** (**A**) Scatterplot showing correlation of the NASF-scores among three replicates of Sec13 immunoprecipitates from HBE41o- cells. (**B**) Protein network interacting with Sec13 in each individual compartment of the cell (nucleus, cytoplasm, mitochondria, ER, cell surface, Golgi, endosomes, secretory proteins, peroxisome and lysosome) (see also **Fig. 5** summary). Here, we recovered known interactions with COPII components which include those contributing to ER autophagy^1-4^, as well as the recognized interactions with NPC^5-9^ and GATOR2 complexes^10-12^ as well as numerous uncharacterized interactors. Our results are consistent with a recent study using proximity labeling to capture Sec13 interactions with a number of cellular components other than the COPII coat machinery^13^. Moreover, and consistent with a more general role as a potential regulator of endomembrane function, we found that Sec13 interacts directly or indirectly with 28 proteins involved in histone modification of which several are directly implicated in histone acetylation and methylation regulating the condensation state of the chromatin impacting chromatin structure and function^14^. In addition, Sec13 tethering to chromatin recruits the chromatin-remodeling complex and is sufficient to induce chromatin decondensation leading to a global as well as gene-specific chromatin changes in transcription^15^. Sec13 is suspected to be the initial NPC component recruited to soon-to-be active genomic sites and in turn recruits other NPCs such as Nup98^9^. Furthermore, Sec13 silencing specifically inhibits transcription of its target genes and prevents efficient reactivation of gene transcription repressed by heat shock. We identified several RNAPII subunits such as POLR2C and POLR2I, for example. These results are supported by the fact that Sec13 has been associated with a large number of genes involved in transcriptional elongation^9,16^ and its recruitment to target sites is accompanied by the recruitment and enrichment of RNA Polymerase II (RNAPII) machinery^9^. Moreover, we identified interactors responsible for RNA maturation and degradation belonging to the spliceosome, the exosome and other RNA processing pathway complexes (**Fig. 5**) as well as in translation of proteins responsible for ribosome biogenesis as well as regulatory factors such as Eif2Bs factors or Eif4As (**Fig. 5**).

In addition to its role in gene expression and translation, evidence from the enriched interactome suggests a more extensive role for Sec13 in endomembrane trafficking beyond its role in COPII assembly at the ER^17,18^ and its role in COPII to COPI exchange at the ERES-ERGIC interface^19-24^. We identified Sec13 interactor proteins localized to early Golgi compartments responsible for glycosylation modifications of N-linked oligosaccharides acquired in the ER, as well as interactions at the trans-Golgi network (TGN) that is responsible for the transport of proteins from the Golgi complex to the cell surface^25^ (**Fig. 5**). Previous efforts in *Arabidopsis* have localized Sec13 at the Golgi and pre-vacuolar compartments, potentially playing a role in vacuolar trafficking by controlling the membrane association of specific proteins^26^. Sec13 has been identified as part of a regulated protein sorting mechanism in the late secretory pathway of *Saccharomyces cerevisiae*, controlling the transport from the Golgi to the plasma membrane^27^. In a related study Sec13 has been suggested to direct docking of COPII carriers to Golgi compartments through PARQ3^28^. Moreover, the interaction of Sec13 with TECPR2 suggests a role for Sec13 in neuronal differentiation and pathophysiology^13^. From a more general perspective, Sec13 has been suggested to be a central feature controlling response to nutrient deprivation^29^ and appears to play a key role in the differential management of mammalian secretion systems^30^.

**Figure S2. Complete Sec13 interactome enrichment.** The 413 proteins comprising the Sec13 core interactome were mapped to the STRING database for cellular component analysis. The Sec13 interactome enrichment is composed of 73 cellular components (STRING enrichment strength between 0.3 & 1; purple) and 22 cellular components highly enriched (STRING enrichment strength > 1; orange).

**Figure S3. HBE41o- proteome profiling enrichment following siRNA-mediated Sec13 silencing.** The 226 (**A**) and 174 (**B**) proteins significantly increased and decreased upon Sec13 depletion, respectively, were mapped to the STRING database for cellular component analysis. The enrichment list is composed of cellular components enriched (STRING enrichment strength between 0.3 and 1; purple) and of cellular components highly enriched (STRING enrichment strength > 1; orange).

**Figure S4.** **Comparison of Sec13 interactors and HBE41o- proteome upon Sec13 silencing.** (**A**) Identification of Sec13 interactors found among the HBE41o- proteome changes upon Sec13 silencing (fold-change < 0.8 or > 1.2). Sec13 Interactors significantly increased or decreased in the HBE41o- proteome upon Sec13 silencing are colored in orange and purple, respectively. (**B**) Details of the comparison between Sec13 interactome data and HBE41o- proteome data showing individual protein names.

**Figure S5. Effect of siRNA silencing on protein COPII component intracellular levels.** (**A,B**) Immunoblot analysis of CFTR, Sar1a, Sar1b, Sec13, Sec31A, Sec23A, Sec23B, Sec24A, Sec24B, Sec24C and Sec24D expression following transfection of (**A**) HBE41o- (WT CFTR) and (**B**) CFBE41o- (F508del CFTR) cells with the independent siRNA targeting the different COPII core subunits. Data are presented with 3 replicates per conditions.

**Figure S6. Sec13 specific involvement in CFTR stability modulation.** (**A**) Immunoblot analysis (left) and quantification (right) of CFTR expression following transfection of HBE41o- cells with two different siSec13 targeting different regions of the Sec13 coding sequence. Data are presented in the right panel as fold-change relative of CFTR band B plus band C to siScr control transfection (mean ± SEM, n≥2). (**B**) Immunoblot analysis of WT CFTR expression following a time-course of Sec13 depletion for 2, 3 and 4 days in HBE41o- cells. (**C**) Immunoblot analysis of CFTR WT and F508del expression following the combined depletion of Sec13 and all Sec24 isoforms compared to siScr control in HBE41o- cells and CFBE41o-, respectively.

**Figure S7. siSec13 does not induces ER stress and does not change CFTR mRNA level.** (**A**) Immunoblot of CFTR, Hsp90, calnexin, GRP94 and ribophorin B expression following siSec13 transfection in HBE410- cells compared to siScr. (**B**) Quantification of CFTR mRNA level following transfection of HBE41o- and CFBE41o- cells with siSec13 compared to siScr. mRNA was standardized by quantification of GUS mRNA, and all values were expressed relative to GUS (mean ± SEM, n ≥ 4).

**Figures S8-10.** Source material Immunoblots shown in **Figs**. **S8-S10** used to build cropped panels shown in **Figs. 2-4** in the main text, respectively. The immunoblots in **Figs.** **S8**, **S9** and **S10** **(panels A-C)** were cut prior to processing (using Ponceau for protein visualization) before probing with primary antibodies allowing us to probe different regions of the same membrane for different proteins so full blots are unavailable. Blots were developed using x-ray film in most cases- therefore, the markers do not appear on all the blots. Membrane edges are not clear in some cases due to high signal-to-noise ratio of luminescence or/and x-ray exposure intensity where applicable. However, shown are complete membrane strips from a given blot reflecting need to separate gel lanes for immunoblotting with specific antibodies. **Fig. S10D** shows the full blots with markers using the probe antibodies 3G11 (upper panel) and Mr. Pink (lower panel). Figure panels **S8A**, **B**, **C**, and **D** show the regions of the blot (red boxes) used to build panels in **Fig 2D**, **E**, **G** and **H**, respectively. Figure panels **S9A** and **B** show the entire blots used to build **Figs**. **3A** and **B**, respectively. Figure panels **S10A**, **B**, **C** and **D** show the regions of the blot (red boxes where applicable) used to build **Figs. 4A**, **B**, **C** and **D**, respectively. All blots are represented in their original aspect ratio within each blot.

**Supplemental Table Legends**

**Table S1. High confidence proteins recovered in the Sec13 interactome.** Co-purifying protein identification technology (CoPIT) coupled with multidimensional chromatography and MS was used to determine the Sec13 interactome in the HBE41o- cell line expressing WT CFTR that were classified as high-confidence interactors.

**Table S2**. **Lower confidence proteins recovered in the Sec13 interactome.** Co-purifying protein identification technology (CoPIT) coupled with multidimensional chromatography and MS^31^ used to determine the Sec13 interactome in HBE41o- cell line harboring WT CFTR that were classified as low-confidence interactors.

**Table S3. Proteins recovered in siSec13 treated WT CFTR expressing HBE41o-**. The relative expression level of proteins in WT expressing HBE41o- cells treated with either siSec13 or a control scrambled siRNA (siScr) using a TMT-based isobaric labeling method followed by data acquisition using MudPIT.

**References cited**

1 Parashar, S. & Ferro-Novick, S. Architecture of the endoplasmic reticulum plays a role in q proteostasis. *Autophagy* **18**, 937-938 (2022).

2 Li, Z., Huang, W. & Wang, W. Multifaceted roles of COPII subunits in autophagy. *Biochim Biophys Acta Mol Cell Res* **1867**, 118627 (2020).

3 Cui, Y., Parashar, S. & Ferro-Novick, S. A new role for a COPII cargo adaptor in autophagy. *Autophagy* **16**, 376-378 (2020).

4 Cui, Y., Parashar, S., Zahoor, M., Needham, P. G., Mari, M., Zhu, M., Chen, S., Ho, H. C., Reggiori, F., Farhan, H., Brodsky, J. L. & Ferro-Novick, S. A COPII subunit acts with an autophagy receptor to target endoplasmic reticulum for degradation. *Science* **365**, 53-60 (2019).

5 Brohawn, S. G., Leksa, N. C., Spear, E. D., Rajashankar, K. R. & Schwartz, T. U. Structural evidence for common ancestry of the nuclear pore complex and vesicle coats. *Science* **322**, 1369-1373 (2008).

6 Enninga, J., Levay, A. & Fontoura, B. M. Sec13 shuttles between the nucleus and the cytoplasm and stably interacts with Nup96 at the nuclear pore complex. *Molecular and cellular biology* **23**, 7271-7284 (2003).

7 Hsia, K.-C., Stavropoulos, P., Blobel, G. & Hoelz, A. Architecture of a coat for the nuclear pore membrane. *Cell* **131**, 1313-1326 (2007).

8 Zhu, X., Huang, G., Zeng, C., Zhan, X., Liang, K., Xu, Q., Zhao, Y., Wang, P., Wang, Q., Zhou, Q., Tao, Q., Liu, M., Lei, J., Yan, C. & Shi, Y. Structure of the cytoplasmic ring of the Xenopus laevis nuclear pore complex. *Science* **376**, eabl8280 (2022).

9 Capelson, M., Liang, Y., Schulte, R., Mair, W., Wagner, U. & Hetzer, M. W. Chromatin-bound nuclear pore components regulate gene expression in higher eukaryotes. *Cell* **140**, 372-383 (2010).

10 Bar-Peled, L., Chantranupong, L., Cherniack, A. D., Chen, W. W., Ottina, K. A., Grabiner, B. C., Spear, E. D., Carter, S. L., Meyerson, M. & Sabatini, D. M. A Tumor suppressor complex with GAP activity for the Rag GTPases that signal amino acid sufficiency to mTORC1. *Science* **340**, 1100-1106 (2013).

11 Parmigiani, A., Nourbakhsh, A., Ding, B., Wang, W., Kim, Y. C., Akopiants, K., Guan, K.-L., Karin, M. & Budanov, A. V. Sestrins inhibit mTORC1 kinase activation through the GATOR complex. *Cell reports* **9**, 1281-1291 (2014).

12 Valenstein, M. L., Rogala, K. B., Lalgudi, P. V., Brignole, E. J., Gu, X., Saxton, R. A., Chantranupong, L., Kolibius, J., Quast, J. P. & Sabatini, D. M. Structure of the nutrient-sensing hub GATOR2. *Nature* **607**, 610-616 (2022).

13 Nalbach, K., Schifferer, M., Bhattacharya, D., Ho-Xuan, H., Tseng, W., Williams, L. A., Stolz, A., Lichtenthaler, S. F., Elazar, Z. & Behrends, C. Spatial proteomics reveals secretory pathway disturbances caused by neuropathy-associated TECPR2. *Nat Commun* **14**, 870 (2023).

14 Ptak, C. & Wozniak, R. W. Nucleoporins and chromatin metabolism. *Current opinion in cell biology* **40**, 153-160 (2016).

15 Kuhn, T. M., Pascual-Garcia, P., Gozalo, A., Little, S. C. & Capelson, M. Chromatin targeting of nuclear pore proteins induces chromatin decondensation. *Journal of Cell Biology* **218**, 2945-2961 (2019).

16 Capelson, M. & Hetzer, M. W. The role of nuclear pores in gene regulation, development and disease. *EMBO reports* **10**, 697-705 (2009).

17 Wang, C., Angles, F. & Balch, W. E. Triangulating variation in the population to define mechanisms for precision management of genetic disease. *Structure* **30**, 1190-1207 e1195 (2022).

18 Anglès, F., Wang, C. & Balch, W. E. Spatial covariance analysis reveals the residue-by-residue thermodynamic contribution of variation to the CFTR fold. *Communications biology* **5**, 1-16 (2022).

19 Rowe, T., Aridor, M., McCaffery, J. M., Plutner, H., Nuoffer, C. & Balch, W. E. COPII vesicles derived from mammalian endoplasmic reticulum microsomes recruit COPI. *J Cell Biol* **135**, 895-911 (1996).

20 Aridor, M., Bannykh, S. I., Rowe, T. & Balch, W. E. Sequential coupling between COPII and COPI vesicle coats in endoplasmic reticulum to Golgi transport. *J Cell Biol* **131**, 875-893 (1995).

21 Ogen-Shtern, N., Chang, C., Saad, H., Mazkereth, N., Patel, C., Shenkman, M. & Lederkremer, G. Z. COP I and II dependent trafficking controls ER-associated degradation in mammalian cells. *iScience* **26**, 106232 (2023).

22 Aridor, M., Fish, K. N., Bannykh, S., Weissman, J., Roberts, T. H., Lippincott-Schwartz, J. & Balch, W. E. The Sar1 GTPase coordinates biosynthetic cargo selection with endoplasmic reticulum export site assembly. *J Cell Biol* **152**, 213-229 (2001).

23 Bannykh, S. I. & Balch, W. E. Membrane dynamics at the endoplasmic reticulum-Golgi interface. *J Cell Biol* **138**, 1-4 (1997).

24 Bannykh, S. I., Rowe, T. & Balch, W. E. The organization of endoplasmic reticulum export complexes. *J Cell Biol* **135**, 19-35 (1996).

25 Zhang, N. & Zabotina, O. A. Critical Determinants in ER-Golgi Trafficking of Enzymes Involved in Glycosylation. *Plants (Basel)* **11** (2022).

26 Lee, M. H., Lee, S. H., Kim, H., Jin, J. B., Kim, D. H. & Hwang, I. A WD40 repeat protein, Arabidopsis Sec13 homolog 1, may play a role in vacuolar trafficking by controlling the membrane association of AtDRP2A. *Molecules & Cells (Springer Science & Business Media BV)* **22** (2006).

27 Roberg, K. J., Rowley, N. & Kaiser, C. A. Physiological regulation of membrane protein sorting late in the secretory pathway of Saccharomyces cerevisiae. *The Journal of cell biology* **137**, 1469-1482 (1997).

28 Cao, Q., Wang, Z., Wan, H., Xu, L., You, X., Liao, L. & Chen, Y. PAQR3 Regulates Endoplasmic Reticulum-to-Golgi Trafficking of COPII Vesicle via Interaction with Sec13/Sec31 Coat Proteins. *iScience* **9**, 382-398 (2018).

29 Kasberg, W., Luong, P., Swift, K. & Audhya, A. Nutrient deprivation alters the rate of COPII coat assembly to tune secretory protein transport. *Res Sq* (2023).

30 Jia, R., Xu, L., Sun, D. & Han, B. Genetic marker identification of SEC13 gene for milk production traits in Chinese holstein. *Front Genet* **13**, 1065096 (2022).

31 Pankow, S., Bamberger, C., Calzolari, D., Martínez-Bartolomé, S., Lavallée-Adam, M., Balch, W. E. & Yates, J. R. ∆ F508 CFTR interactome remodelling promotes rescue of cystic fibrosis. *Nature* **528**, 510-516 (2015).
