# Supplementary material for: COPII cage assembly factor Sec13 integrates information flow regulating endomembrane function in response to human variation
Source: Sci Rep. 2024 May 3;14:10160. doi: 10.1038/s41598-024-60687-2 (PMC11065896; doi:10.1038/s41598-024-60687-2)
Supplement: Supplementary file 3 — Supplementary Figures. [file 41598_2024_60687_MOESM3_ESM.pdf]

Figure S1

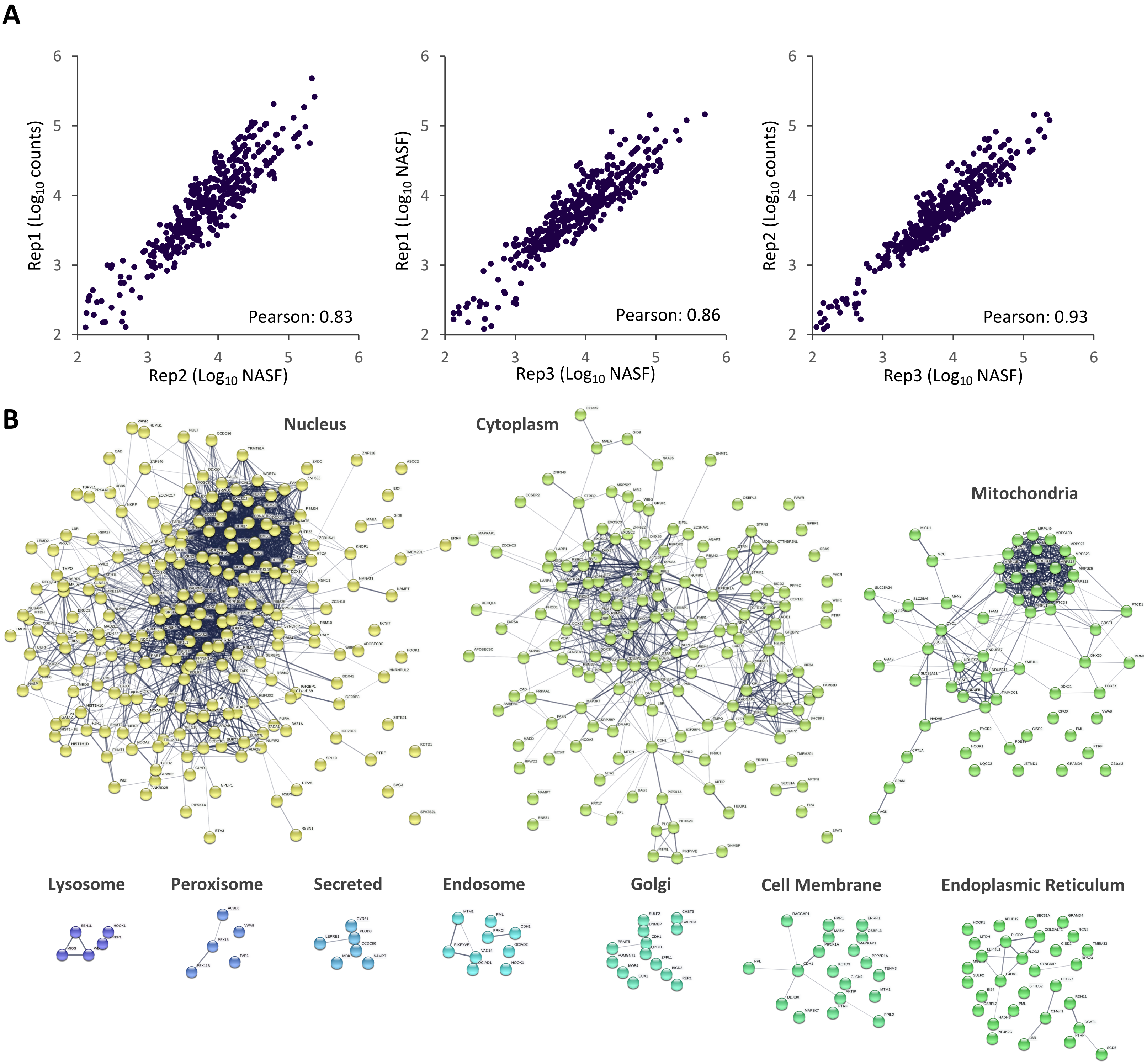
A
Rep1 (Log10 counts)
Rep2 (Log10 NASF)
Pearson: 0.83
Rep1 (Log10 NASF)
Rep3 (Log10 NASF)
Pearson: 0.86
Rep2 (Log10 counts)
Rep3 (Log10 NASF)
Pearson: 0.93
2
3
4
5
6
B
Nucleus
Cytoplasm
Mitochondria
Lysosome
Peroxisome
Secreted
Endosome
Golgi
Cell Membrane
Endoplasmic Reticulum

Figure S2

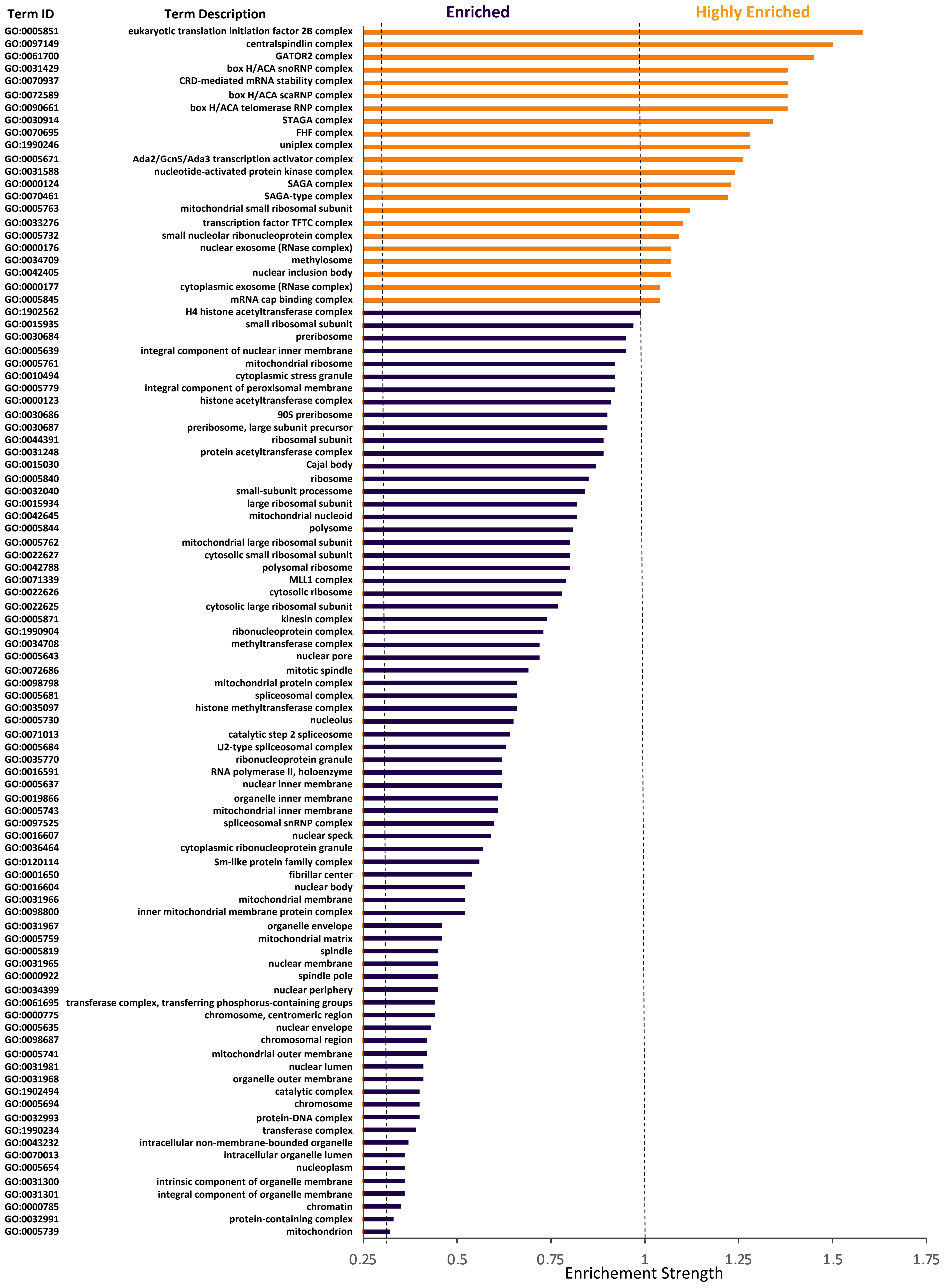
Term ID
Term Description
Enriched
Highly Enriched
GO:0005851 eukaryotic translation initiation factor 2B complex
GO:0097149 centralspindlin complex
GO:0061700 GATOR2 complex
GO:0031429 box H/ACA snoRNP complex
GO:0070937 CRD-mediated mRNA stability complex
GO:0072589 box H/ACA scaRNP complex
GO:0090661 box H/ACA telomerase RNP complex
GO:0030914 STAGA complex
GO:0070695 FHF complex
GO:1990246 uniplex complex
GO:0005671 Ada2/Gcn5/Ada3 transcription activator complex
GO:0031588 nucleotide-activated protein kinase complex
GO:0000124 SAGA complex
GO:0070461 SAGA-type complex
GO:0005763 mitochondrial small ribosomal subunit
GO:0033276 transcription factor TFTC complex
GO:0005732 small nucleolar ribonucleoprotein complex
GO:0000176 nuclear exosome (RNase complex)
GO:0034709 methylosome
GO:0042405 nuclear inclusion body
GO:0000177 cytoplasmic exosome (RNase complex)
GO:0005845 mRNA cap binding complex
GO:1902562 H4 histone acetyltransferase complex
GO:0015935 small ribosomal subunit
GO:0030684 preribosome
GO:0005639 integral component of nuclear inner membrane
GO:0005761 mitochondrial ribosome
GO:0010494 cytoplasmic stress granule
GO:0005779 integral component of peroxisomal membrane
GO:0000123 histone acetyltransferase complex
GO:0030686 90S preribosome
GO:0030687 preribosome, large subunit precursor
GO:0044391 ribosomal subunit
GO:0031248 protein acetyltransferase complex
GO:0015030 Cajal body
GO:0005840 ribosome
GO:0032040 small-subunit processome
GO:0015934 large ribosomal subunit
GO:0042645 mitochondrial nucleoid
GO:0005844 polysome
GO:0005762 mitochondrial large ribosomal subunit
GO:0022627 cytosolic small ribosomal subunit
GO:0042788 polysomal ribosome
GO:0071339 MLL1 complex
GO:0022626 cytosolic ribosome
GO:0022625 cytosolic large ribosomal subunit
GO:0005871 kinesin complex
GO:1990904 ribonucleoprotein complex
GO:0034708 methyltransferase complex
GO:0005643 nuclear pore
GO:0072686 mitotic spindle
GO:0098798 mitochondrial protein complex
GO:0005681 spliceosomal complex
GO:0035097 histone methyltransferase complex
GO:0005730 nucleolus
GO:0071013 catalytic step 2 spliceosome
GO:0005684 U2-type spliceosomal complex
GO:0035770 ribonucleoprotein granule
GO:0016591 RNA polymerase II, holoenzyme
GO:0005637 nuclear inner membrane
GO:0019866 organelle inner membrane
GO:0005743 mitochondrial inner membrane
GO:0097525 spliceosomal snRNP complex
GO:0016607 nuclear speck
GO:0036464 cytoplasmic ribonucleoprotein granule
GO:0120114 Sm-like protein family complex
GO:0001650 fibrillar center
GO:0016604 nuclear body
GO:0031966 mitochondrial membrane
GO:0098800 inner mitochondrial membrane protein complex
GO:0031967 organelle envelope
GO:0005759 mitochondrial matrix
GO:0005819 spindle
GO:0031965 nuclear membrane
GO:0000922 spindle pole
GO:0034399 nuclear periphery
GO:0061695 transferase complex, transferring phosphorus-containing groups
GO:0000775 chromosome, centromeric region
GO:0005635 nuclear envelope
GO:0098687 chromosomal region
GO:0005741 mitochondrial outer membrane
GO:0031981 nuclear lumen
GO:0031968 organelle outer membrane
GO:1902494 catalytic complex
GO:0005694 chromosome
GO:0032993 protein-DNA complex
GO:1990234 transferase complex
GO:0043232 intracellular non-membrane-bounded organelle
GO:0070013 intracellular organelle lumen
GO:0005654 nucleoplasm
GO:0031300 intrinsic component of organelle membrane
GO:0031301 integral component of organelle membrane
GO:0000785 chromatin
GO:0032991 protein-containing complex
GO:0005739 mitochondrion
0.25
0.5
0.75
1
1.25
1.5
1.75
Enrichement Strength

B

Figure S3A-increased

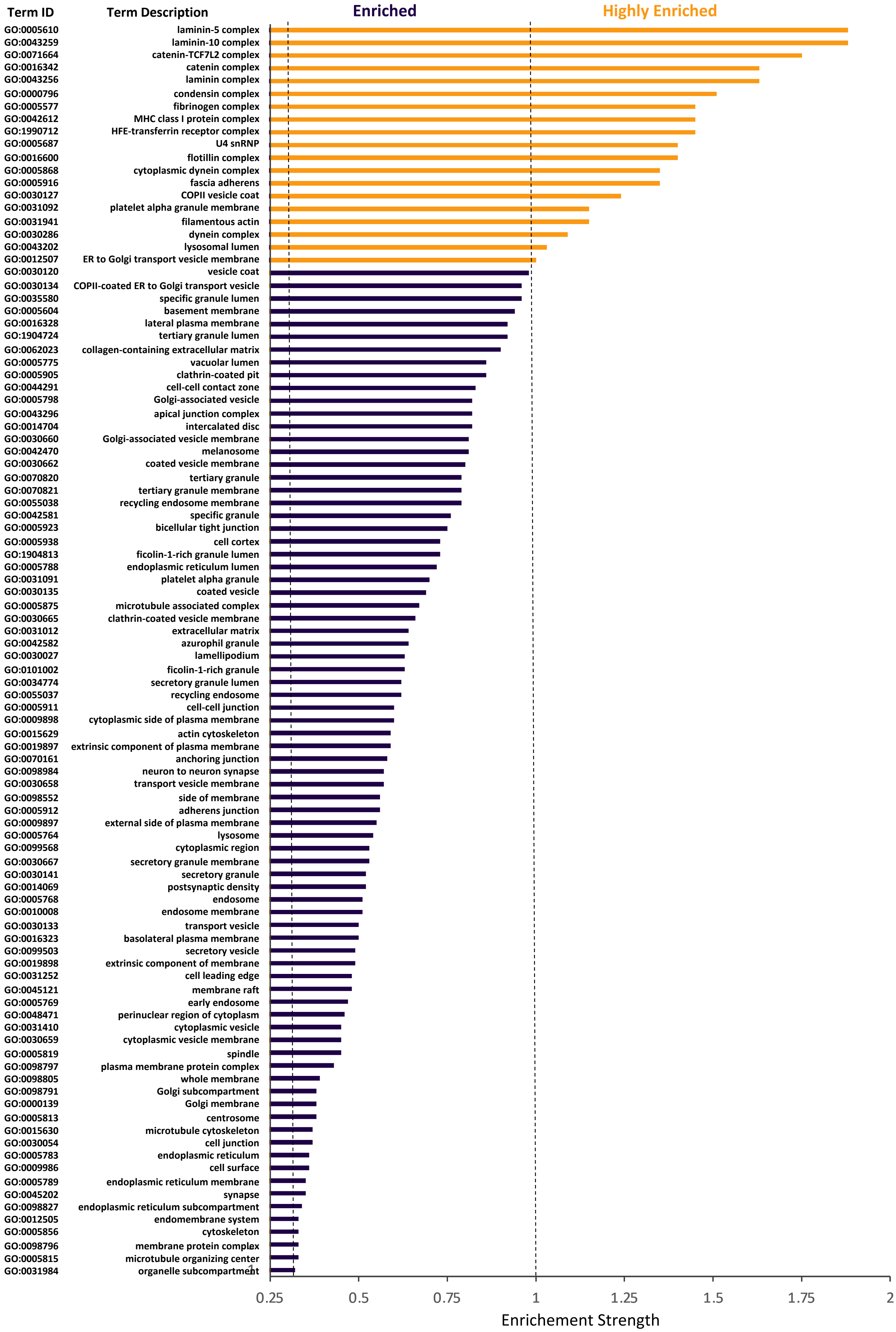

Figure S3B-decreased

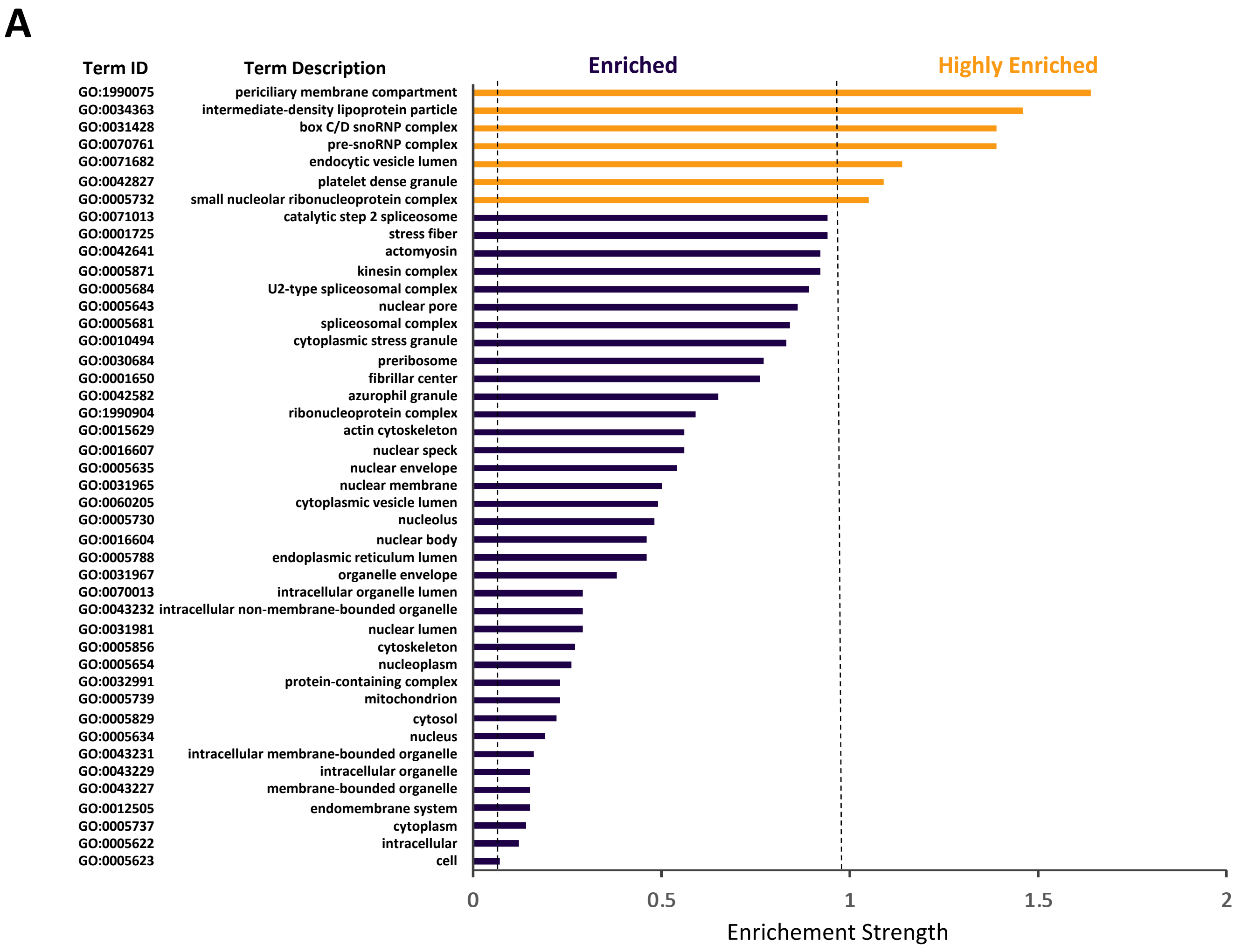

Figure S4

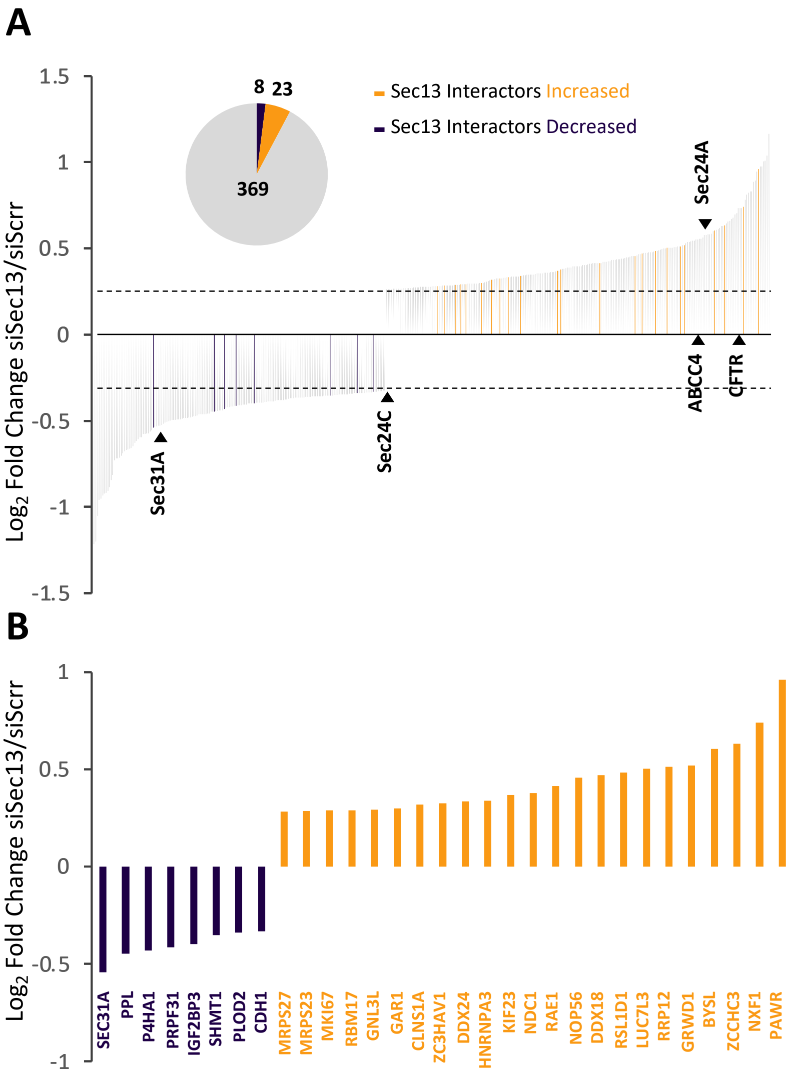
A
1.5
1
0.5
0
-0.5
-1
-1.5
Log2 Fold Change siSec13/siScrr
8
23
369
Sec13 Interactors Increased
Sec13 Interactors Decreased
Sec24A
ABCC4
CFTR
Sec31A
Sec24C
B
1
0.5
0
-0.5
-1
Log2 Fold Change siSec13/siScrr
SEC31A
PPL
P4HA1
PRPF31
IGF2BP3
SHMT1
PLOD2
CDH1
MRPS27
MRPS23
MKI67
RBM17
GNL3L
GAR1
CLNS1A
ZC3HAV1
DDX24
HNRNPA3
KIF23
NDC1
RAE1
NOP56
DDX18
RSL1D1
LUC7L3
RRP12
GRWD1
BYSL
ZCCHC3
NXF1
PAWR

Figure S5

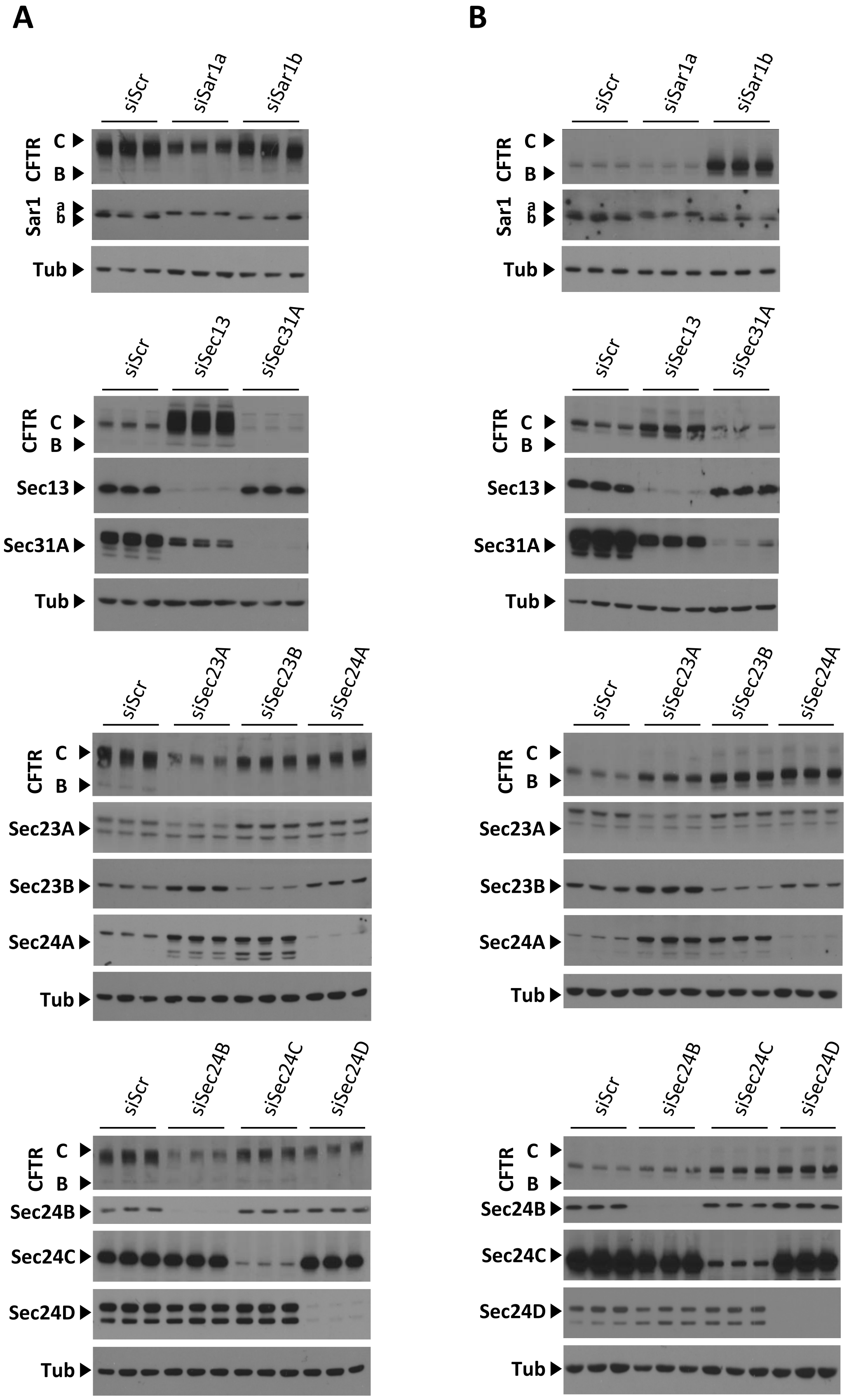
A
B
siScr
siSar1a
siSar1b
CFTR
C
B
Sar1
a
b
Tub
siSec13
siSec31A
Sec13
Sec31A
siSec23A
siSec23B
siSec24A
Sec23A
Sec23B
Sec24A
siSec24B
siSec24C
siSec24D
Sec24B
Sec24C
Sec24D

Figure S6

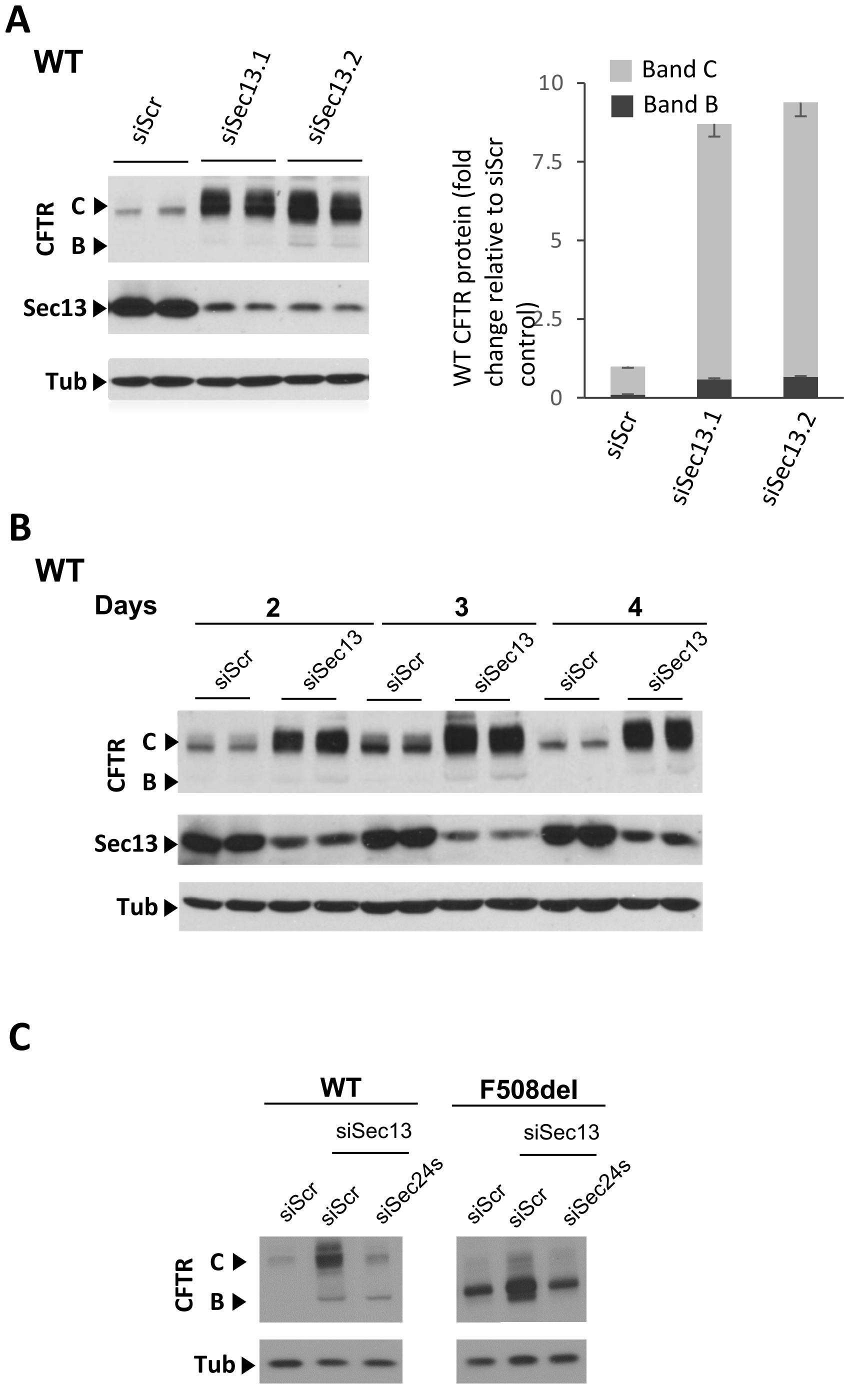
A
WT
siScr
siSec13.1
siSec13.2
CFTR
C
B
Sec13
Tub
Band C
Band B
WT CFTR protein (fold change relative to siScr control)
10
7.5
5
2.5
0
siScr
siSec13.1
siSec13.2
B
WT
Days
2
3
4
siScr
siSec13
siScr
siSec13
siScr
siSec13
CFTR
C
B
Sec13
Tub
C
WT
F508del
siSec13
siSec13
siScr
siScr
siSec24s
siScr
siScr
siSec24s
CFTR
C
B
Tub

Figure S7

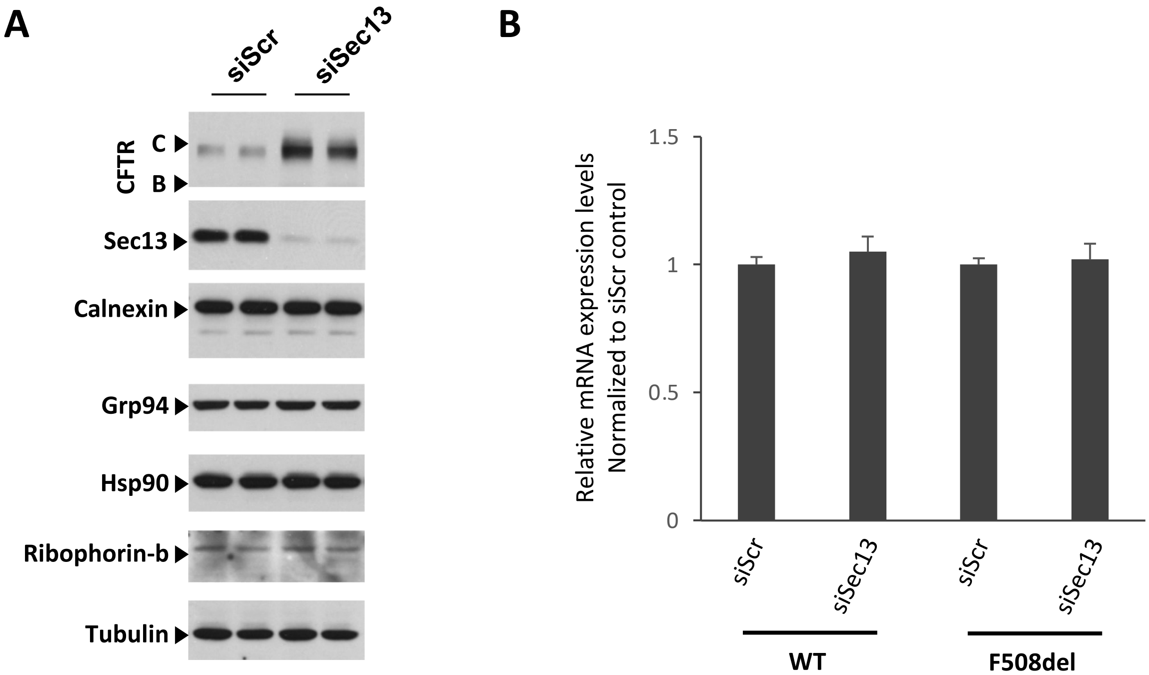
A
siScr
siSec13
CFTR
C
B
Sec13
Calnexin
Grp94
Hsp90
Ribophorin-b
Tubulin
B
Relative mRNA expression levels
Normalized to siScr control
1.5
1
0.5
0
siScr
siSec13
siScr
siSec13
WT
F508del

Figure S8

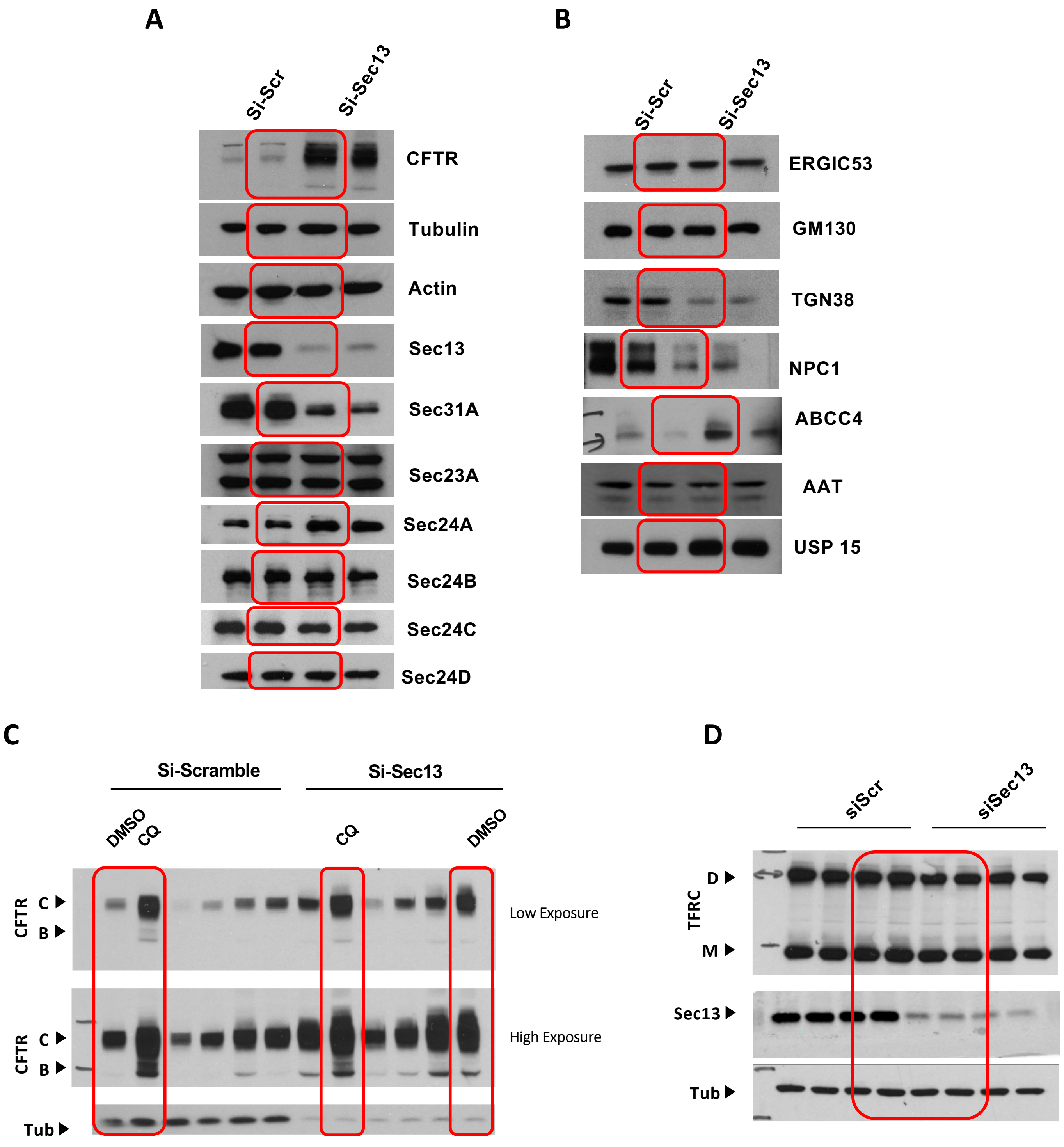
A
Si-Scr
Si-Sec13
CFTR
Tubulin
Actin
Sec13
Sec31A
Sec23A
Sec24A
Sec24B
Sec24C
Sec24D
B
Si-Scr
Si-Sec13
ERGIC53
GM130
TGN38
NPC1
ABCC4
AAT
USP 15
C
Si-Scramble
Si-Sec13
DMSO
CQ
CQ
DMSO
CFTR
C
B
Low Exposure
CFTR
C
B
High Exposure
Tub
D
siScr
siSec13
TFRC
D
M
Sec13
Tub

Figure S9

A

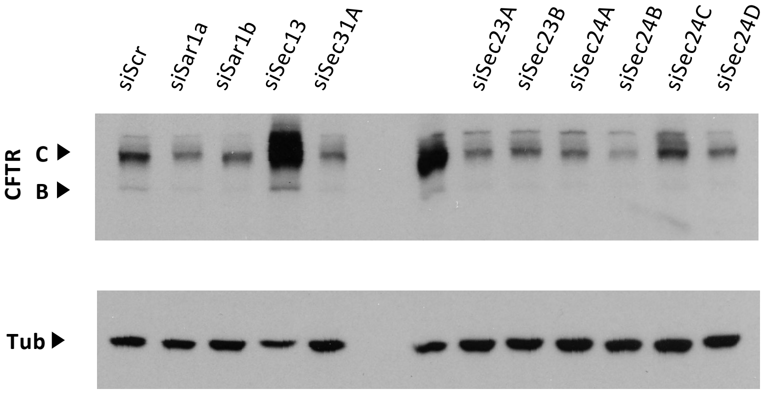
siScr
siSar1a
siSar1b
siSec13
siSec31A
siSec23A
siSec23B
siSec24A
siSec24B
siSec24C
siSec24D
CFTR
C
B
Tub

B

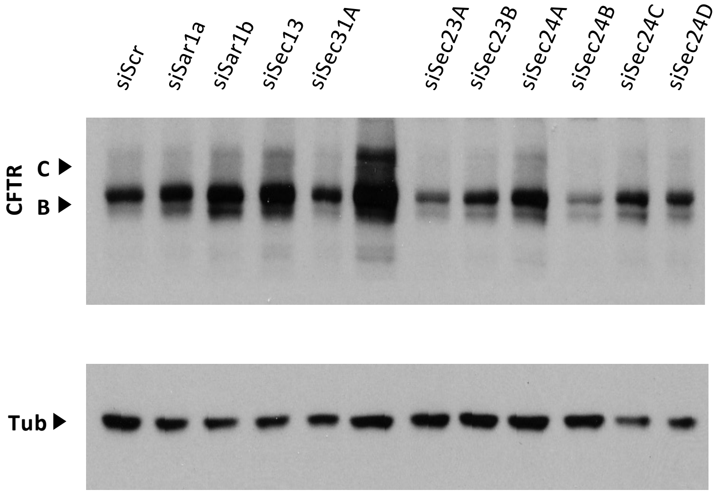
siScr
siSar1a
siSar1b
siSec13
siSec31A
siSec23A
siSec23B
siSec24A
siSec24B
siSec24C
siSec24D
CFTR
C
B
Tub

Figure S10

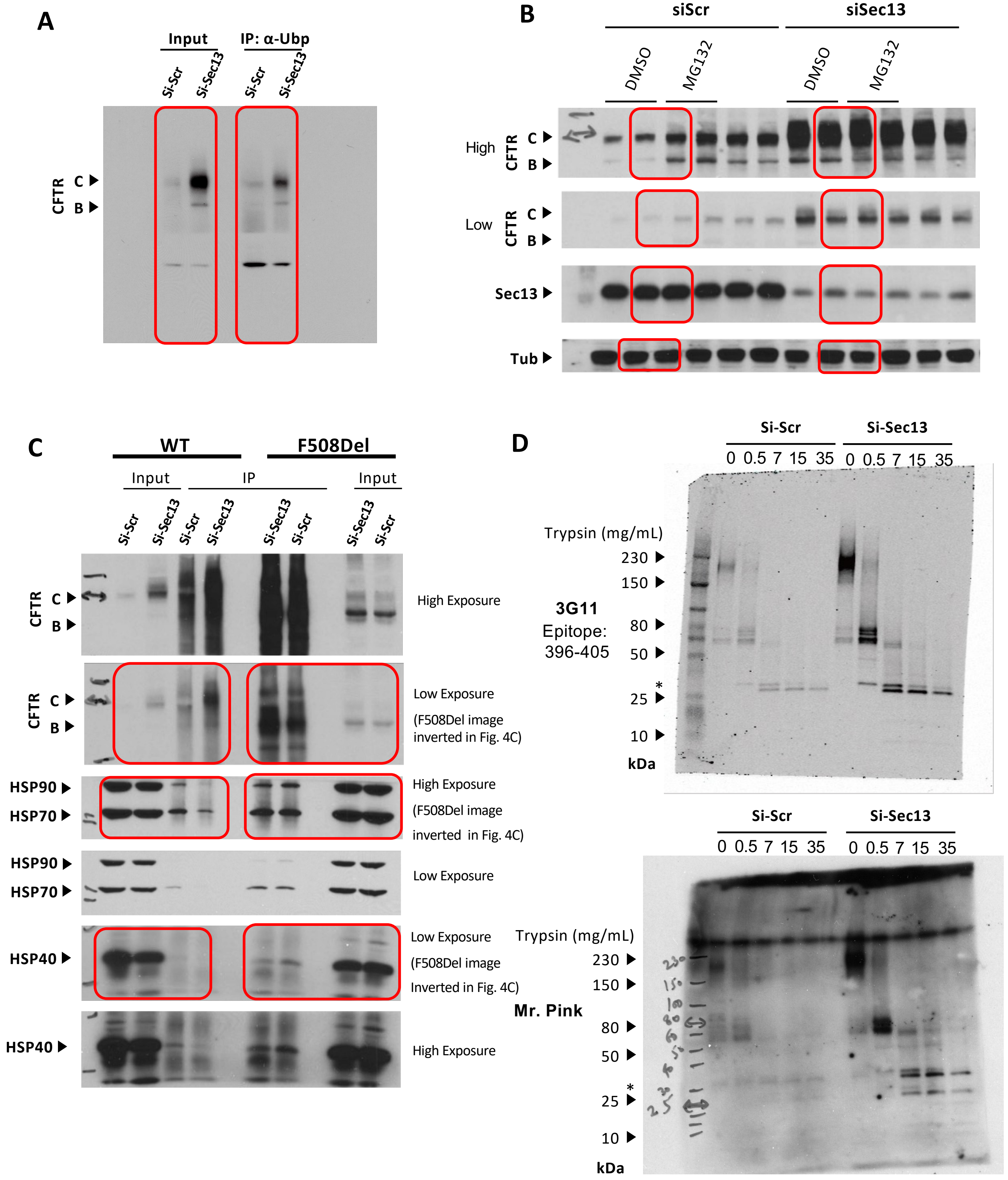

A
Input
IP: α-Ubp
Si-Scr
Si-Sec13
Si-Scr
Si-Sec13
CFTR
C
B
B
siScr
siSec13
DMSO
MG132
DMSO
MG132
High
Low
CFTR
C
B
Sec13
Tub
C
WT
F508Del
Input
IP
Input
Si-Scr
Si-Sec13
Si-Scr
Si-Sec13
Si-Sec13
Si-Scr
Si-Sec13
Si-Scr
CFTR
C
B
High Exposure
Low Exposure
(F508Del image inverted in Fig. 4C)
HSP90
HSP70
High Exposure
(F508Del image inverted in Fig. 4C)
Low Exposure
HSP40
Low Exposure
(F508Del image Inverted in Fig. 4C)
HSP40
High Exposure
D
Si-Scr
Si-Sec13
0 0.5 7 15 35
0 0.5 7 15 35
Trypsin (mg/mL)
3G11
Epitope: 396-405
230
150
80
50
*
25
10
kDa
Si-Scr
Si-Sec13
0 0.5 7 15 35
0 0.5 7 15 35
Trypsin (mg/mL)
Mr. Pink
230
150
80
50
*
25
10
kDa
